# Stability of Studtite in Saline Solution: Identification of Uranyl–Peroxo–Halo Complex

**DOI:** 10.1021/acs.inorgchem.2c00233

**Published:** 2022-05-24

**Authors:** Junyi Li, Zoltán Szabó, Mats Jonsson

**Affiliations:** Department of Chemistry, School of Engineering Sciences in Chemistry, Biotechnology and Health, KTH Royal institute of Technology, SE-10044 Stockholm, Sweden

## Abstract

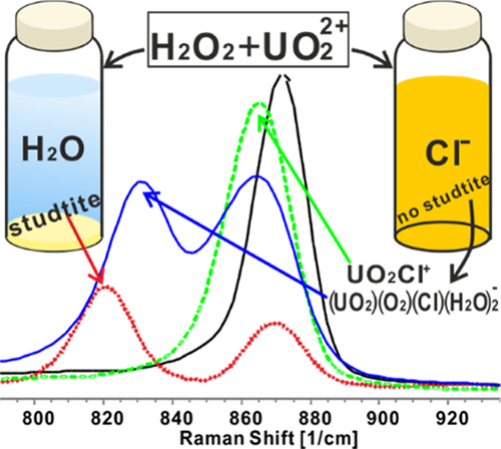

Hydrogen peroxide is produced upon radiolysis of water and has been shown to be the main oxidant driving oxidative dissolution of UO_2_-based nuclear fuel under geological repository conditions. While the overall mechanism and speciation are well known for granitic groundwaters, considerably less is known for saline waters of relevance in rock salt or during emergency cooling of reactors using seawater. In this work, the ternary uranyl–peroxo–chloro and uranyl–peroxo–bromo complexes were identified using IR, Raman, and nuclear magnetic resonance (NMR) spectroscopy. Based on Raman spectra, the estimated stability constants for the identified uranyl–peroxo–chloro ((UO_2_)(O_2_)(Cl)(H_2_O)_2_^–^) and uranyl–peroxo–bromo ((UO_2_)(O_2_)(Br)(H_2_O)_2_^–^) complexes are 0.17 and 0.04, respectively, at ionic strength ≈5 mol/L. It was found that the uranyl–peroxo–chloro complex is more stable than the uranyl–peroxo–bromo complex, which transforms into studtite at high uranyl and H_2_O_2_ concentrations. Studtite is also found to be dissolved at a high ionic strength, implying that this may not be a stable solid phase under very saline conditions. The uranyl–peroxo–bromo complex was shown to facilitate H_2_O_2_ decomposition via a mechanism involving reactive intermediates.

## Abstract 2

Aqueous solutions containing UO_2_^2+^ and H_2_O_2_ are stabilized by the presence of chloride. This is attributed to the formation of uranyl−chloro and uranyl−peroxo−chloro complexes preventing the precipitation of studtite. The existence of these complexes was confirmed using IR, Raman, and NMR spectroscopies.

## Introduction

One of the major challenges of the nuclear industry is to handle the highly radiotoxic used nuclear fuel after removal from a nuclear reactor. Several strategies for handling the used fuel have been proposed, but only two have been developed to a mature state. One strategy is to reprocess the used fuel and recover material that can be used to produce new fuel, and the other strategy is the permanent placement of the used nuclear fuel in a geological repository. In the latter case, natural and engineered barriers will prevent the radionuclides from the used nuclear fuel from reaching the biosphere before the radioactivity of the fuel has reached levels corresponding to the specific activity of a naturally occurring uranium ore (in the order of 10^5^ years). Reprocessing will also generate highly radioactive waste, but since this is generally more short-lived, a repository for this radioactive material does not have to maintain barrier integrity for the same extended period of time. A key issue for the design, construction, and licensing of a geological repository for radioactive waste is to perform reliable safety assessments. A scenario that has to be addressed in such a safety assessment is groundwater intrusion as a consequence of multiple barrier failure.

Used or spent commercial nuclear fuel is composed of 95% UO_2_, and the remaining fraction consists of fission products and heavier actinides.^1−3^ The fuel matrix, UO_2_, has very low solubility in the slightly reducing groundwaters found at the planned locations of several repositories. However, radiolysis of groundwater adjacent to the fuel surface, caused by the inherent radioactivity of the fuel, will produce oxidizing (HO^•^, HOO^•^, and H_2_O_2_) as well as reducing (e_aq_^–^, H^•^, and H_2_) species.^4,5^ For kinetic reasons, the oxidants will dominate the surface reactions initially and oxidize the UO_2_ matrix from U(IV) to considerably more soluble U(VI) and thereby enable dissolution of the fuel matrix and the subsequent release of the highly radiotoxic fission products and heavy actinides into the biosphere.^6,7^ Oxidized UO_2_ is released as uranyl (UO_2_^2+^), which is a good electron acceptor in solution, and therefore tends to coordinate with Lewis base ligands. It is well known that UO_2_^2+^ can form complexes and clusters with a wide variety of Lewis base ligands such as water, carbonate, hydroxide, peroxide, and halides.^8−10^ Vallet et al.^11^ reported the affinity of the ligands to increase in the order H_2_O < Cl^–^ < F^–^ < OH^–^ < CO_3_^2–^ < O_2_^2–^ based on quantum chemical calculations. UO_2_^2+^ binding to O_2_^2–^ in aqueous solution at low concentrations of competing ligands can precipitate as studtite at ambient temperature and as meta-studtite at temperatures higher than 70 °C.^12,13^ Studtite and meta-studtite are the only two uranyl peroxide minerals found in nature,^14^ and they have also been found on the surface of spent nuclear fuel,^15^ on the “lava” of the Chernobyl disaster,^16^ and on damaged reactor cores after the Fukushima nuclear accident.^17^

In addition to uranyl peroxides, investigations of complexes formed between UO_2_^2+^ and chloride and bromide in aqueous systems have also received renewed attention. In particular, the chloro complexes are important in the speciation of uranium in the biosphere and dissolution of the UO_2_ fuel matrix under certain conditions.^18,19^ Nguyen-Trung et al.^10^ and Takao et al.^20^ reported that the relative strength of uranyl halide complexes follows the order I^–^ < Br^–^ < Cl^–^ < F^–^. In addition, Grenthe et al.^21^ investigated the coordination of UO_2_^2+^ with Cl^–^ and Br^–^ in aqueous systems. It was shown that uranyl and chloride can form uranyl monochloro complex UO_2_Cl^+^ with the logarithmic gross stability constants log β_1_ = 0.17 ± 0.02 at zero ionic strength and uranyl dichloro complex UO_2_Cl_2_ (log β_2_ = −1.1 ± 0.4, *I* = 0). However, Br^–^ was found to only form monobromo complex UO_2_Br^+^ with UO_2_^2+^ (log β_1_ = 0.22 ± 0.02), at zero ionic strength. In addition, Soderholm et al.^22^ also studied the coordination between UO_2_^2+^ and Cl^–^ in aqueous system at a constant ionic strength of 5.3 m. It was reported that the maximum number of coordinated Cl^–^ is 3 and the stability constants were found to be β_1_ = 1.5 ± 0.01, β_2_ = 0.8 ± 0.04, and β_3_ = 0.4 ± 0.01.

Uranyl halide complexes are of practical importance in mainly two situations: (1) If seawater is used for emergency cooling of the core of a damaged nuclear reactor (e.g., in the Fukushima nuclear accident after which 1.25 million tonnes of seawater have been pumped through the damaged units to prevent the molten fuel debris from overheating, and pumping continues.^23−25^ The concentration of Cl^–^ has been reported as 0.6 M in the pacific ocean near Fukushima^26^); (2) If a deep geological repository for spent nuclear fuel is placed in rock salt as a host rock. This is an option that has been considered by several countries.^27−29^ Upon water intrusion following barrier failure, the spent nuclear fuel would come in contact with highly saline water. In both situations, uranyl–chloro complexes may play an important role. According to Hata et al.^30^ the radiation chemical yield of H_2_O_2_ in γ radiolysis of water is only marginally affected at Cl^–^ concentrations below 1 M (decreases by 10% at 0.6 M Cl^–^).

In a very recent work, the H_2_O_2_-induced oxidative dissolution of UO_2_ in saline aqueous solutions was partly explained by the formation of ternary uranyl–peroxo–chloro and uranyl–peroxo–bromo complexes.^19^ The existence of ternary uranyl–peroxo complexes is well established in carbonate-containing solutions, where uranyl–peroxo–carbonate complexes are formed and in alkaline solutions where uranyl–peroxo–hydroxo complexes can be formed.^31,32^ Previous studies have shown that studtite and meta-studtite can dissolve and transform into the ternary complexes under these conditions.^12^ Given the indirect indication of the existence of ternary uranyl–peroxo–halo complexes and their potential importance in understanding spent nuclear fuel dissolution in saline solutions, it is essential to investigate their possible existence and properties using more direct methods. According to a DFT study by Odoh and Schreckenbach,^33^ the uranyl–peroxo–fluoro complex could possibly exist, but to the best of our knowledge, no experimental or theoretical work has reported the existence of the corresponding chloro or bromo complexes.

In this work, we have explored the possible existence and properties of uranyl–peroxo–chloro/bromo complexes in aqueous solution using IR (ATR-IR), TIR-Raman, and NMR (^35^Cl NMR and ^17^O NMR) spectroscopies in combination with time-resolved measurements of uranium and hydrogen peroxide concentrations in solution.

## Experimental Procedures

All solutions were prepared using Milli-Q water (18.2 MΩ cm), and all chemicals used were of reagent grade unless otherwise stated. Uranyl nitrate (UO_2_(NO_3_)_2_·6H_2_O, Merck), hydrogen peroxide 30% (Merck), and sodium halide salts including NaCl (Sigma-Aldrich), NaBr (Acros Organics), NaClO_4_ (Sigma-Aldrich), as well as sodium bicarbonate (NaHCO_3_, Merck) were used to prepare stock solutions, which were then diluted to the desired concentrations of UO_2_^2+^, H_2_O_2_, Cl_,_^–^ Br^–^, ClO_4_^–^, and HCO_3_^–^, respectively.

The concentration of H_2_O_2_ was measured using the Ghormley triiodide method, where I^–^ is first oxidized to I_3_^–^ by H_2_O_2_, and then the absorbance of I_3_^–^ was measured by UV/vis spectrophotometry (λ = 360 nm).^34,35^ The concentration of U(VI) in solution was measured using the Arsenazo III method, where uranyl reacts with the Arsenazo III reagent forming a stable complex in acidic media. The absorbance of the formed complex was measured at λ = 653 nm by UV/vis spectrophotometry.^36^ Potassium iodide (Merck) and Arsenazo III (Sigma-Aldrich) were used in the Ghormley triiodide method and Arsenazo III method. The analysis was performed in duplicate for each measurement of H_2_O_2_ and U(VI) concentration. The difference between the duplicate measurements was less than 2.3 and 0.82 μM for H_2_O_2_ and U(VI), respectively. All experiments were performed at least three times. The error bars in the figures reflect the results of these experiments and are based on the standard deviation derived from the three repetitions of each experiment.

### Studtite Formation Dynamics in Halide Solutions

UO_2_^2+^ and H_2_O_2_ (both 0.2 mM) were added to aqueous solutions of different concentrations of halide and perchlorate (from 10 mM to 1 M) in a total volume of 25 mL. The concentrations of UO_2_^2+^ and H_2_O_2_ were measured as a function of time for 24 h. The pH of the solution was recorded before and after each experiment.

### XRD (for the Precipitates Formed in Halide Solutions)

The precipitates formed in the solution after mixing 20 mM H_2_O_2_ and 20 mM U(VI) in 2 M NaCl or NaBr solution for 5 days was first dried in air and then characterized using powder X-ray diffraction (XRD). XRD patterns were recorded at room temperature using a PANalytical X’Pert PRO diffractometer using a Bragg–Brentano geometry and Cu Kα radiation (1.5418 Å) in a 2θ range between 10 and 80°. The powder sample was ground manually in an agate mortar.

### Spectroscopic Studies of the Formation of Uranyl Complexes

The complexes formed in solutions containing 20 mM UO_2_^2+^ in H_2_O and 5 M Cl^–^ or Br^–^ with and without the presence of 20 mM H_2_O_2_ were characterized by ATR-IR, Raman, and NMR spectroscopies.

### IR and Raman Spectroscopies

The IR spectra were collected in a Fourier transform infrared (FT-IR) spectrometer (Nicole iS10, Thermo Scientific). The universal diamond attenuated total reflectance (ATR) sampling accessory was used for all of the measurements. The IR resolution in the IR spectra is <4 cm^–1^. The Raman spectra were obtained with a home-built spectrometer.^37^ The laser source is a highly stable CW laser with a wavelength of 532 nm (Laser Quantum, U.K.). An external polarizer and a half-wave plate were used to precisely control the polarization of the incident laser beam. The Raman scattered light is collected using an ultralong working distance objective (M-Plan Apo 50X, N.A. 0.55, Mitotoyo, Japan) with a 90° angle configuration. The objective is attached to a modified upright Axio microscope (Zeiss, Germany). The Raman scattered light is then passed through a long-pass filter (Semrock-Razor Edge), an achromatic half-wave plate, and a polarizer (Thorlabs), before being focused to a spectrograph slit (Shamrock-Andor, Ireland) and detected by a CCD camera (Newton 940-Andor, Ireland). Since the scattered light can be unpolarized, even if the excitation source is polarized depending on the type of vibration, the second polarizer ensures that the polarization of the scattered light entering the spectrograph slit is always vertical (linearly polarized). The half-wave plate changes the polarization component of the scattered light between parallel (*I*_||_) and perpendicular (*I*_⊥_) with respect to the polarization direction of the incident laser beam. The *I*_||_ polarization combination was used for all Raman measurements in this work. Only liquid samples were measured in this work. These samples were put in a 0.7 mL glass vial and sealed with an aluminum cap (VWR). During the measurement, the incident laser was focused on the bulk of the solution.

### ^35^Cl NMR and ^17^O NMR

The ^35^Cl (39.2 MHz) and ^17^O (54.2 MHz) NMR spectra were recorded at 298 K on a Bruker DMX-400 spectrometer using a 10 mm normal broadband probe. The probe temperature was measured by a calibrated Pt-100 resistance thermometer and adjusted using a Bruker Eurotherm variable-temperature control unit. The samples were prepared by adding 10% v/v D_2_O for lock and were transferred to 10 mm NMR tubes. The samples for ^17^O NMR experiments were prepared from ^17^O-enriched uranyl nitrate stock solution. It was prepared by dissolving UO_2_(NO_3_)_2_·6H_2_O in ^17^O-enriched water (Isotec, Inc., 29.0% ^17^O) in a quartz cuvette followed by UV irradiation overnight. The process resulted in ca. 15% ^17^O isotope enrichment of the uranyl site when isotope equilibrium was reached. The ^35^Cl spectra are referenced to the ^35^Cl signal of 5 M NaCl in H_2_O (in the presence of 10% v/v D_2_O), while the ^17^O NMR spectra are referenced to ^17^O NMR signal of tap water, both measured at 298 K.

## Results and Discussion

As uranyl halide complexes are always considered to be weak complexes, speciation calculations were performed for systems containing 0–5 M Cl^–^ or Br^–^ with 0.2 or 20 mM UO_2_^2+^ using equilibrium constants of uranyl–hydroxo and uranyl–chloro or −bromo complexes^21^ (Figures S1–S4 and Table S1). The calculations show that the uranyl–chloro/bromo complexes are the dominating species in the solutions at high Cl^–^ or Br^–^ concentrations, respectively.

### Time-Resolved Studies of [UO_2_^2+^] and [H_2_O_2_] in Aqueous Solutions

**Figure 1:**
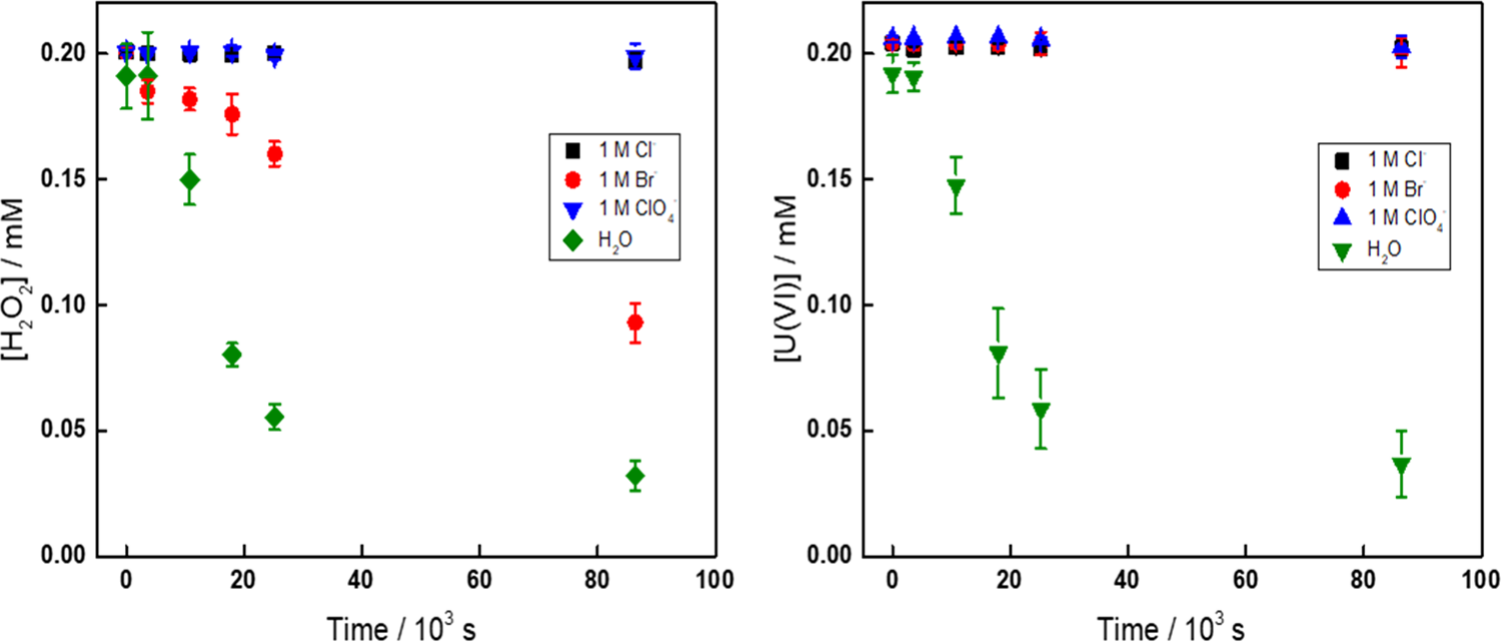
Concentrations of H_2_O_2_ (left) and UO_2_^2+^ (right) in 1 M Cl^–^, Br^–^, ClO_4_^–^ solutions and H_2_O as a function of time.

The stability of H_2_O_2_ and UO_2_^2+^ in aqueous solutions containing 0.2 mM UO_2_^2+^ and 0.2 mM H_2_O_2_ was studied as a function of Cl^–^, Br^–^, and ClO_4_^–^ concentrations, respectively. The concentrations were varied from 10 mM to 1 M. The results were compared to a reference sample with H_2_O as a solvent. The pH values for all of the samples were measured before and after the experiments (shown in Table S2). In Figure 1, the results for 1 M Cl^–^, Br^–^, and ClO_4_^–^ are presented together with the reference sample.

As can be seen in Figure 1, the concentrations of UO_2_^2+^ and H_2_O_2_ decrease fairly rapidly in the reference sample. During the experiment, the solution becomes hazy and a precipitate is formed. As has been shown in several previous studies, the precipitate is studtite.^17,38−40^ In solutions containing 1 M Cl^–^ and 1 M ClO_4_^–^, neither the UO_2_^2+^ nor the H_2_O_2_ concentration change with time, indicating that studtite formation is prevented by both ions. As ClO_4_^–^ is not expected to form complexes with UO_2_^2+^, the stabilizing effect of Cl^–^ and ClO_4_^–^ must be attributed to an ionic strength effect. As an increased ionic strength would facilitate particle aggregation and precipitation, the observed ionic strength effect must be attributed to processes prior to nucleation. In studtite, negatively charged peroxo groups link the positively charged uranyl ions and we speculate that the kinetics of the linking process becomes slower at increasing ionic strength as the process involves attractive electrostatic forces. In the solution containing 1 M Br^–^, it is evident that the UO_2_^2+^ concentration remains constant during the experiment, while the H_2_O_2_ concentration is decreasing. The fact that only the H_2_O_2_ concentration decreases during the experiment indicates that studtite is not formed and the H_2_O_2_ consumption must be attributed to another process. It is interesting to note that the same behavior has been observed in solutions containing H_2_O_2_, UO_2_^2+^, and HCO_3_^–^.^12^ The nature of this process will be further discussed later on.

The same type of experiment was repeated at 10, 50, 100, 250, and 500 mM Cl^–^, Br^–^, and ClO_4_^–^, respectively. The results of these experiments are shown in the Supporting Information (Figures S5–S9). In general, the stability of the solutions decreases with decreasing ionic strength. For the solutions containing Cl^–^ and ClO_4_^–^, the decrease in UO_2_^2+^ concentration parallels the decrease in H_2_O_2_ concentration, which indicates that studtite formation is taking place. In most cases, the concentration is initially stable but starts to decrease within a few hours. This could possibly be attributed to slow nucleation. The experiments also show that the stability of the solutions decreases with decreasing concentrations of Cl^–^ or ClO_4_^–^ and no statistically significant differences can be observed between the two anions.

**Figure 2:**
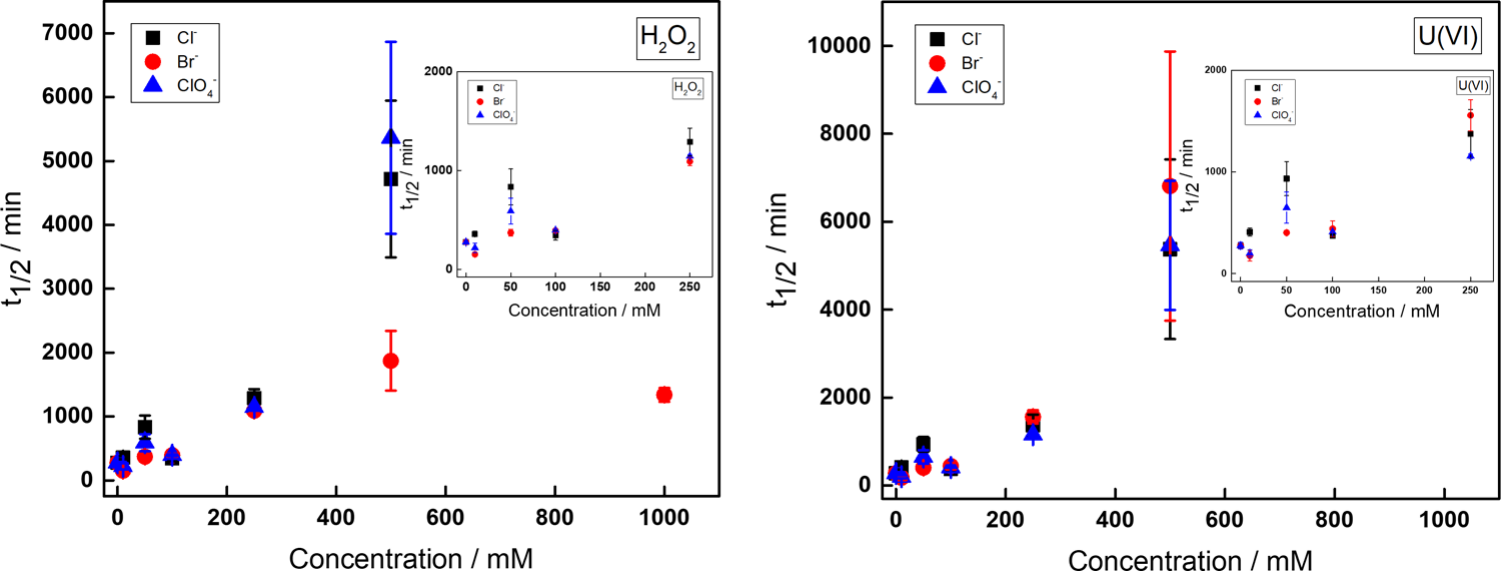
Half-lives of H_2_O_2_ (a) and UO_2_^2+^ (b) in solutions containing 0.2 mM H_2_O_2_ and 0.2 mM UO_2_^2+^ with different concentrations of Cl^–^, Br^–^, and ClO_4_^–^.

To get a better overview, the half-lives of UO_2_^2+^ and H_2_O_2_ are plotted as a function of anion concentration in Figure 2.

Judging from how the half-life of UO_2_^2+^ changes with anion concentration, the effects of Cl^–^, Br^–^, and ClO_4_^–^ are more or less identical. However, when looking at the half-life for H_2_O_2_, it becomes evident that Br^–^ differs from Cl^–^ and ClO_4_^–^ at higher concentrations. At 0.5 M Br^–^ and higher, the half-life of H_2_O_2_ is considerably shorter compared to solutions containing Cl^–^ and ClO_4_^–^. At lower concentrations, the half-lives are comparable for all anions, which indicates that the main process consuming H_2_O_2_ is studtite formation and the process decomposing H_2_O_2_ is of minor importance.

The consumption of H_2_O_2_ in solutions with high Br^–^ concentrations where the concentration of UO_2_^2+^ appears to be stable implies that the presence of Br^–^ and UO_2_^2+^ can catalyze the decomposition of H_2_O_2_. A control experiment with 0.2 mM H_2_O_2_ and 1 M Br^–^ (without UO_2_^2+^) showed that Br^–^ alone does not catalyze the reaction. Hence, both UO_2_^2+^ and Br^–^ are required for this reaction to occur.

**Figure 3:**
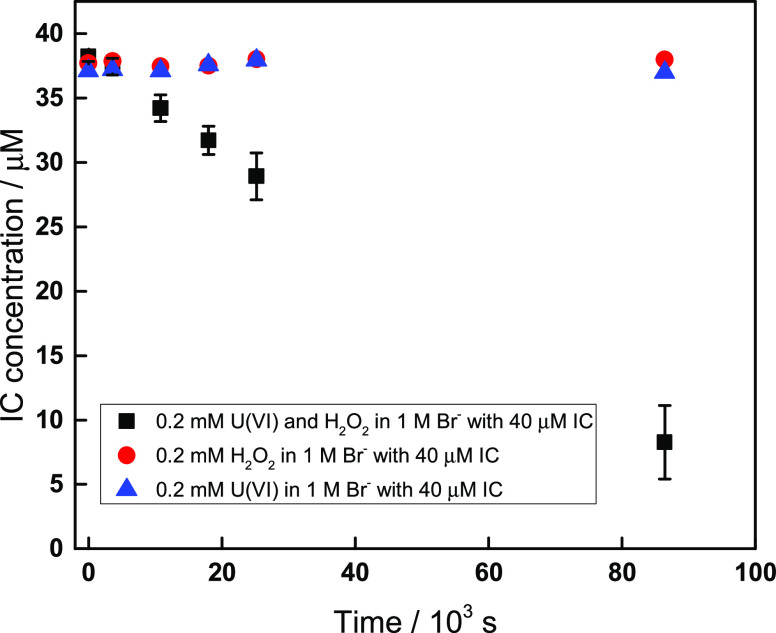
Indigo carmine concentration as a function of time in solutions containing (1) 0.2 mM UO_2_^2+^, 0.2 mM H_2_O_2_, 1 M Br^–^, and 40 μM indigo carmine (black square); (2) 0.2 mM H_2_O_2_, 1 M Br^–^, and 40 μM indigo carmine (red circle); and (3) 0.2 mM UO_2_^2+^, 1 M Br^–^, and 40 μM indigo carmine (blue triangle).

Catalytic decomposition of H_2_O_2_ could possibly involve more reactive intermediates assuming that the O–O bond is being broken in this process. If hydroxyl radicals are produced as an intermediate in this system, they would most likely react with Br^–^ and produce Br_2_^•–^ at the very high Br^–^ concentrations used here. Br_2_^•–^ is a strong one-electron oxidant that can be detected indirectly. Indigo carmine is a dye that has been used before to quantify the formation of reactive oxidants (radicals).^41,42^ In a series of experiments we used 40 μM indigo carmine to probe the possible formation of reactive intermediates. Indigo carmine was added to solutions containing 1 M Br^–^, 0.2 mM H_2_O_2_, and 0.2 mM U(VI). In addition, two control experiments were performed with 40 μM indigo carmine. One control experiment was a solution containing only 1 M Br^–^ and 0.2 mM H_2_O_2_, and the other was a solution containing only 1 M Br^–^ and 0.2 mM U(VI) in addition to the indigo carmine. The concentration of indigo carmine was measured spectrophotometrically at 610 nm and as a function of time. The results are shown in Figure 3. The pH values for the solution containing 0.2 mM H_2_O_2_, 0.2 mM U(VI), and 40 μM indigo carmine in 1 M Br^–^ are 3.6 and 3.9 before and after the experiment, respectively.

As can be seen, indigo carmine is consumed only in the solution containing 1 M Br^–^, 0.2 mM H_2_O_2_, and 0.2 mM UO_2_^2+^, but not in any of the control experiments. Therefore, it is reasonable to state that the catalytic decomposition of H_2_O_2_ in solutions containing UO_2_^2+^ and Br^–^ proceeds via the formation of hydroxyl radicals (OH^•^) or hydroperoxyl radical (HO_2_^•^), as the latter has also been shown to be capable of oxidizing indigo carmine.^41^ As adding uranyl will lead to a decrease in pH, and therefore it is possible that the observed decomposition of H_2_O_2_ is acid-catalyzed rather than UO_2_^2+^-catalyzed.^43^ For this reason, we performed an additional experiment, in which we adjust pH using HCl for the solution without adding uranyl to the same pH value as the solution adding uranyl. The results are shown in Table S3. As can be seen, the H_2_O_2_ decomposition rate in the sample containing 1 M NaBr acidified by HCl is approximately the same as for the sample only containing 1 M NaBr. Hence, we can rule out acid catalysis as the reason for the observed H_2_O_2_ decomposition. Interestingly, H_2_O_2_ decomposition has also been observed in systems containing H_2_O_2_/UO_2_^2+^/HCO_3_^–^.^12^ As H_2_O_2_ decomposition is not observed in systems containing H_2_O_2_/UO_2_^2+^/Cl^–^, we conclude that the presence of UO_2_^2+^ in combination with a more easily oxidized ligand like Br^–^ or CO_3_^2–^ is a prerequisite for catalytic decomposition to occur. It is also interesting to note that the rate of H_2_O_2_ decomposition is higher at 1 M Br^–^ than at 0.5 M, i.e., there is a Br^–^ concentration dependence (as shown in Figure 2). A plausible mechanism for this process would be: H_2_O_2_ + UO_2_^2+^ + Br^–^ → UO_2_(O_2_)Br^–^ + 2H^+^ → OH^•^ + OH^–^ + UO_2_Br^2+^. Since Br^–^ is in excess, it is reasonable to assume the reaction is followed by UO_2_Br^2+^ + 2Br^–^ → UO_2_Br^+^ + Br_2_^•–^.

**Figure 4:**
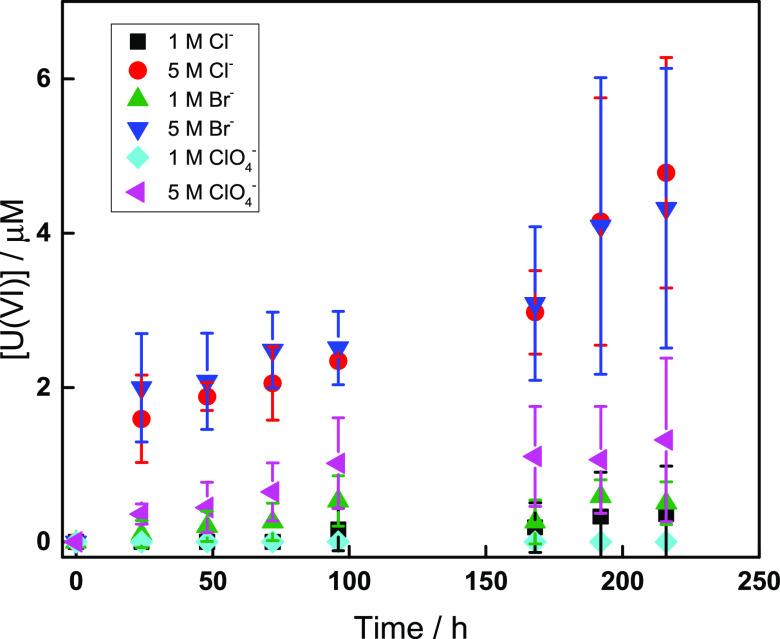
Uranyl concentration as a function of time for aqueous studtite powder suspensions containing 1 M or 5 M Cl^–^, Br^–^, and ClO_4_^–^.

As the results presented above show that high ionic strength stabilizes UO_2_^2+^ and H_2_O_2_ in solution by preventing the formation of solid studtite, we decided to also study the stability of solid studtite in solutions containing 1 and 5 M of NaCl, NaBr, and NaClO_4_. Both the H_2_O_2_ and the UO_2_^2+^ concentrations were monitored as a function of time over a period of 10 days. Since H_2_O_2_ is quite sensitive to a number of external factors, no conclusive trends could be obtained from the H_2_O_2_ concentration variations. UO_2_^2+^ is not as sensitive to external factors, and therefore we can discuss the studtite stability from these data. The UO_2_^2+^ concentrations are plotted against time for the different experiments in Figure 4. Every experiment was repeated three times. The pH values for all of the samples were measured before and after the experiments (shown in Table S2).

In general, the UO_2_^2+^ concentrations are low, but it is quite clear that the higher salt concentration has a stronger impact on the dissolution of studtite than the lower salt concentration. More importantly, UO_2_^2+^ is dissolved more rapidly in solutions containing NaCl and NaBr than in solutions containing NaClO_4_ at both 1 and 5 M. However, the difference is more pronounced at 5 M. This indicates that this is not only an ionic strength effect. The higher rate of dissolution in NaCl and NaBr compared to NaClO_4_ could be attributed to the formation of complexes with UO_2_^2+^ in addition to the ionic strength effect, which is also observed in the solution containing NaClO_4_. While a high ionic strength alone stabilizes solutions containing UO_2_^2+^ and H_2_O_2_ as reflected by the absence of studtite formation, the observed effect of Br^–^ and Cl^–^ in the studtite dissolution experiments may simply be a kinetic effect attributed to surface complex formation.

### Vibrational Spectroscopy

Vibrational spectroscopies, such as IR or Raman spectroscopy, are suitable techniques to characterize species containing UO_2_^2+^, as previous investigations have shown that UO_2_^2+^ displays strong active vibrational bands associated with the covalent axial bonds (O=U=O)^2+^.^10,44−50^ Specifically, one of the three fundamental vibration modes of UO_2_^2+^, the symmetric stretch (ν_1_), is Raman-active, whereas the other two modes, bend (ν_2_) and antisymmetric stretch (ν_3_), are IR-active. From Raman spectra, an increase in the ν_1_ frequency will be observed when H_2_O is substituted by other inorganic ligands in uranyl–aquo complexes, and similarly, an increase in the ν_3_ frequency will be observed when the substitution reactions occur in the uranyl–hydrate complex

To obtain detectable concentrations of the uranyl complexes, higher concentrations of UO_2_^2+^ and H_2_O_2_ must be used (higher than 20 mM). Under these conditions, the solutions are not stable even at a high ionic strength (see sample photographs at different times, Figures S10–S15) although the stability of the solutions is still ionic strength-dependent (more stable at high ionic strength). The precipitate formed is identified by XRD as studtite (Figures S16 and S17).^51^ For this reason, IR and Raman spectra were collected immediately using freshly prepared solutions. No precipitation was observed during the measurement unless otherwise stated. For clarity, Table 1 lists the ranges of concentrations of U(VI), H_2_O_2_, and salts used in various characterizing techniques and the time-resolved studies. The pH values for all of the samples were measured after sample preparation (shown in Table S2).

**Table 1:** Concentrations of U(VI), H_2_O_2_ and Salts Used in Present Work

	[U(VI)] (mM)	[H_2_O_2_] (mM)	salt used in experiments
time-monitoring experiments	0.2	0.2	10/50/100/250/500/1000 mM Cl^–^, Br^–^, and ClO_4_^–^
dissolution experiments	no added	no added	1/5 M Cl^–^, Br, and ClO_4_^–^
Raman	20	20	5 M Cl^–^/Br^–^
IR	20/60	20/40	5 M Cl^–^/Br^–^
^35^Cl NMR	20	20	5 M Cl^–^, 200 mM HCO_3_^–^
^17^O NMR	20	20	5 M Cl^–^/Br^–^, 200 mM HCO_3_^–^

#### Raman Spectroscopy

**Figure 5:**
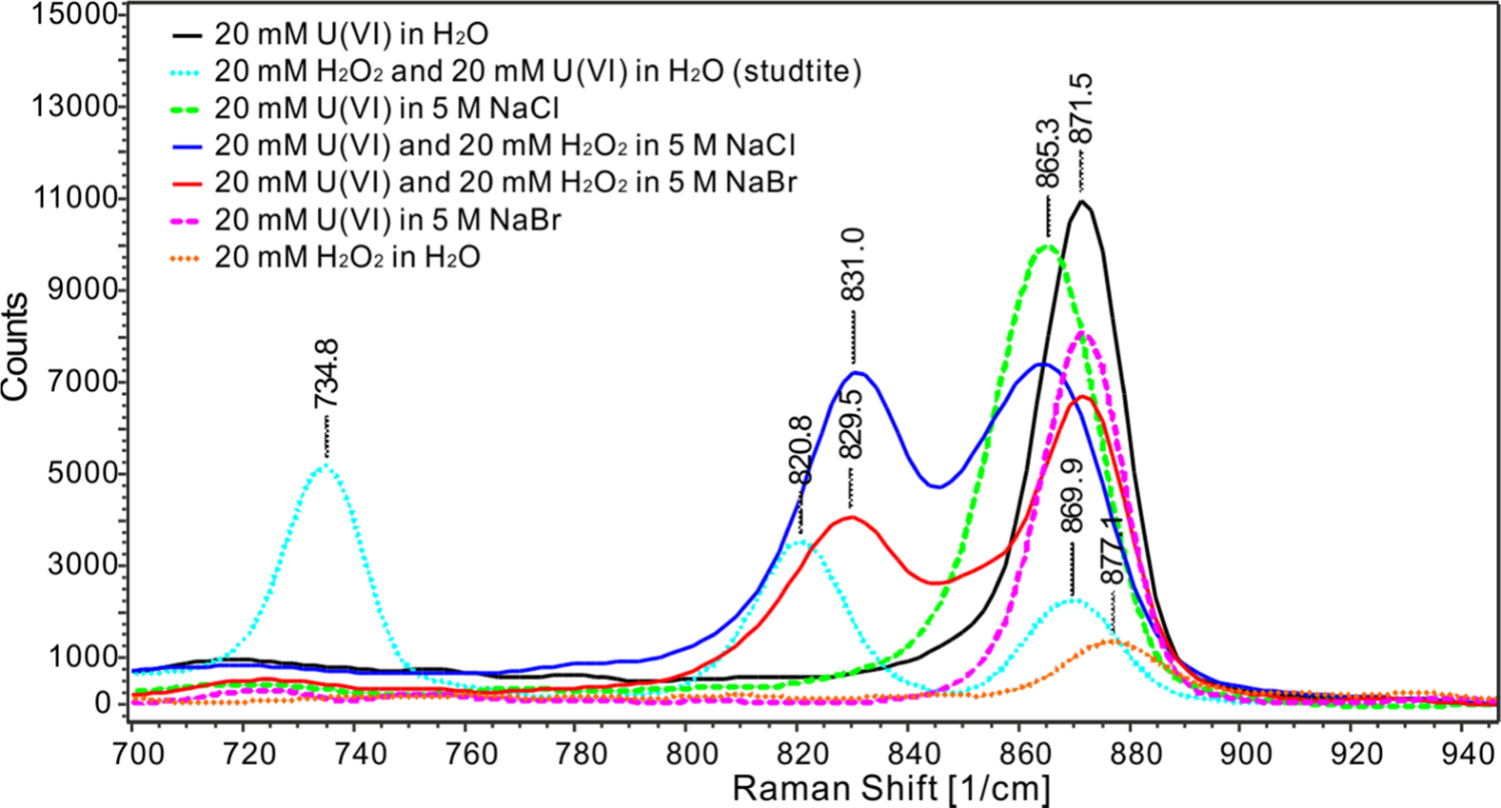
Raman spectra of aqueous solutions containing UO_2_^2+^, H_2_O_2_, NaBr, or NaCl.

**Figure 6:**
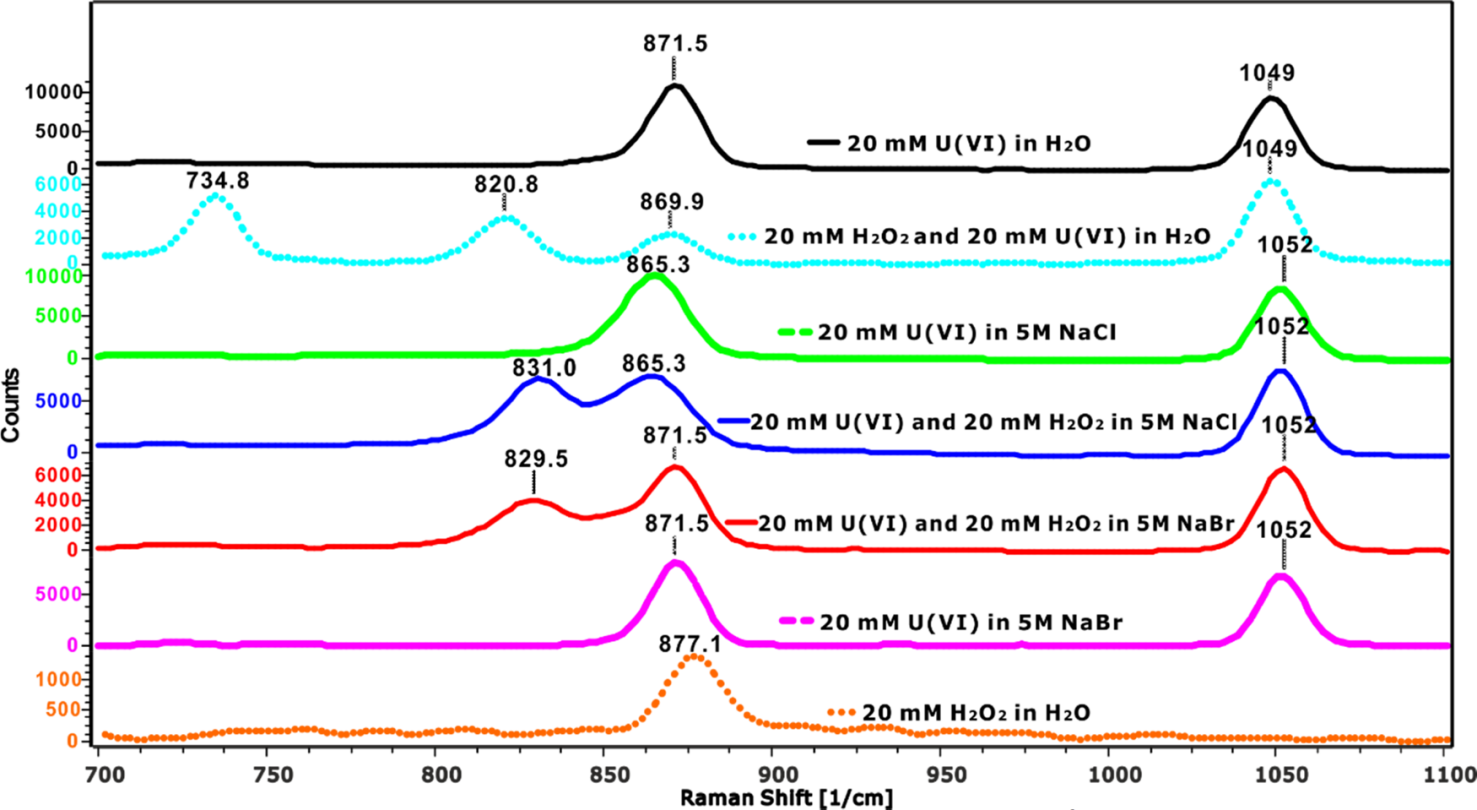
Raman spectra of aqueous solutions containing UO_2_^2+^, H_2_O_2_, NaBr, or NaCl (stacked plot).

As can be seen in Figures 5 and 6, the solution containing only 20 mM uranyl nitrate (black line) has a peak at 1049 cm^–1^, which is attributed to NO_3_^–^, in line with that reported in the literature.^52,53^ This peak shifts to 1052 cm^–1^ at a high ionic strength (i.e., in solutions containing 5 M NaCl or 5 M NaBr). The solution containing only uranyl nitrate also displays a peak at 871.5 cm^–1^, which can be attributed to the uranyl–aquo complex. This is also in good agreement with the literature (870,^10^ 871,^54^ 872 cm^–1^ ^55^). According to X-ray scattering experiments and computational studies, the penta–aquo complex UO_2_(H_2_O)_5_^2+^ is the dominating uranyl–aquo species in this system.^48^ For the solution containing only 20 mM H_2_O_2_ (orange dots), a weak peak appears at 877.1 cm^–1^. This is indicative of the stretching of the O–O bond.^56,57^ In the solution containing 20 mM U(VI) and 20 mM H_2_O_2_ (sky blue dots), a studtite suspension is formed. The Raman spectrum for this system has one uranyl–aquo complex peak and two new peaks at 821 and 735 cm^–1^. The peak at 821 cm^–1^ is in agreement with the spectrum for solid studtite found in the literature,^17,39,58,59^ and the peak at 735 cm^–1^ indicates amorphous uranyl peroxide.^58^ The solution containing 20 mM U(VI) and 5 M NaCl (green dashes) shows one peak at 865.3 cm^–1^ but no peak at 871.5 cm^–1^, which indicates that the uranyl–aquo complex is quantitatively converted into a complex between U(VI) and Cl^–^. According to previous studies, the peak at 865.3 cm^–1^ is attributed to the disubstituted uranyl–chloro complex UO_2_Cl^+^.^10^ In the solution containing 20 mM U(VI) and 5 M NaBr (pink dashes), only a peak at 871 cm^–1^ is observed. A previous study has reported that a monocoordinated uranyl–bromo complex could exist under these conditions and display a Raman peak that overlaps with that of the uranyl–aquo complex.^10^ The solution containing 20 mM H_2_O_2_, 20 mM U(VI), and 5 M NaCl (blue line) shows one peak at 865.3 cm^–1^, indicating the presence of the uranyl–chloro complex and one new peak at 831 cm^–1^ that has not been reported before and that cannot be attributed to any of the individual constituents of the solution. We therefore attribute this peak to a uranyl–peroxo–chloro complex. Interestingly, the solution containing 20 mM H_2_O_2_ and 20 mM U(VI) and 5 M NaBr (red line) displays a new peak in addition to the one observed in the solution containing only 20 mM U(VI) and 5 M NaBr. The new peak occurs at 829.5 cm^–1^, which is quite close to the peak that was attributed to a uranyl–peroxo–chloro complex. This serves as an indication of the existence of a uranyl–peroxo–bromo complex. Judging from the relative peak heights within the Br^–^ and Cl^–^ systems containing H_2_O_2_, the uranyl–peroxo–bromo complex is weaker than the uranyl–peroxo–chloro complex. All peaks measured in Raman are summarized in Table S4.

#### IR Spectroscopy

**Figure 7:**
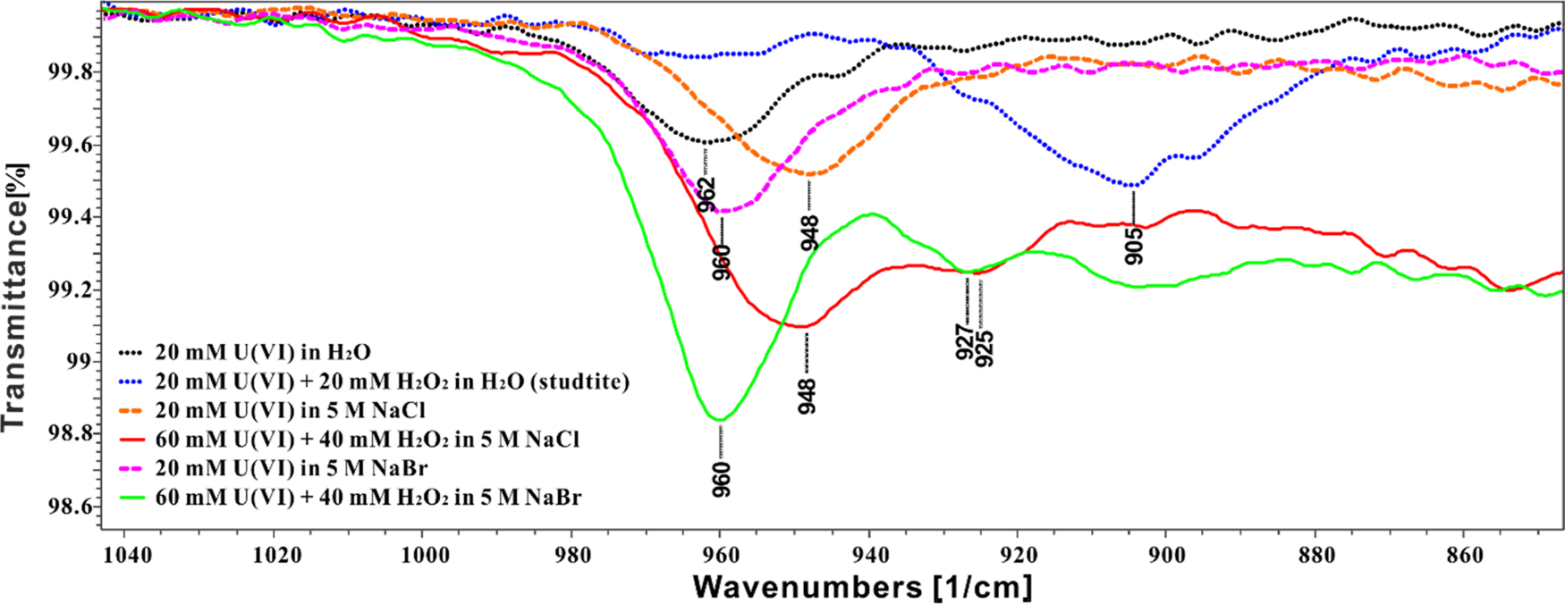
IR spectra of aqueous solutions containing UO_2_^2+^, H_2_O_2_, NaBr, or NaCl.

As can be seen from Figure 7, the solution containing only 20 mM uranyl nitrate (black dots) has a peak at 962 cm^–1^, which is attributed to the uranyl–aquo complex. This is in good agreement with the literature.^48,60^ The spectra for 20 mM H_2_O_2_ in water, in 5 M NaCl, and in 5 M NaBr were collected. These spectra are not shown in Figure 7 because no clear peak can be found in the region of 800–1000 cm^–1^. In the solution containing 20 mM U(VI) and 20 mM H_2_O_2_ (blue dots), a studtite suspension is formed with a peak at 905 cm^–1^. The peak position is close to previously reported solid-phase studtite samples (906,^61^ 909,^59^ and 915 cm^–1^ ^58^). The solution containing 20 mM U(VI) and 5 M NaCl (orange dashes) shows one peak at 948 cm^–1^, indicating the uranyl–chloro complex close to the peak for UO_2_Cl^+^ at 956 cm^–1^ reported previously.^62^ Moreover, in the solution containing 20 mM U(VI) and 5 M NaBr (pink dashes), the solution shows one peak at 960 cm^–1^, which is separated from the uranyl–aquo complex peak (962 cm^–1^); therefore, we conclude that the peak at 960 cm^–1^ in the IR spectrum can be attributed to the uranyl–bromo complex. The solution containing 40 mM H_2_O_2_, 60 mM U(VI), and 5 M NaCl (blue line) shows two peaks at 948 and 925 cm^–1^ attributed to the uranyl–chloro complex and uranyl–peroxo–chloro complex, respectively. The solution containing 40 mM H_2_O_2_ and 60 mM U(VI) and 5 M NaBr (red line) also displays two peaks at 960 and 927 cm^–1^ representing the uranyl–bromo and uranyl–peroxo–bromo complexes. All peaks measured in IR are summarized in Table S5.

### NMR Spectroscopy

#### ^35^Cl NMR

The applications of different dynamic NMR methods are very well documented in the literature; hence, only a short summary relevant to the recent results is given here.^63^ In the so-called slow-exchange region, separate peaks for the exchanging sites can be observed in the spectra. If the rate is too slow to have an effect on the line shape, one- and two-dimensional magnetization transfer experiments could provide kinetic information on the exchanging system. When the exchange rate is fast enough to affect the line shapes, but still too slow on the chemical shift scale to result in a coalescence of peaks, the exchange rate can be calculated from the linewidths of the exchanging species. If the exchange rate is fast on the chemical shift scale (i.e., when the exchange rate is faster than the chemical shift difference of the exchanging sites in Hertz), only one peak can be observed for the exchanging species, as a result of coalescence of the peaks. In this case, the shape of the exchange averaged signal can be calculated by the individual chemical shifts and the relative populations of the exchanging species by a special matrix formalism. Then, the kinetic parameters can be determined by a comparison of the measured and the calculated spectra.

**Figure 8:**
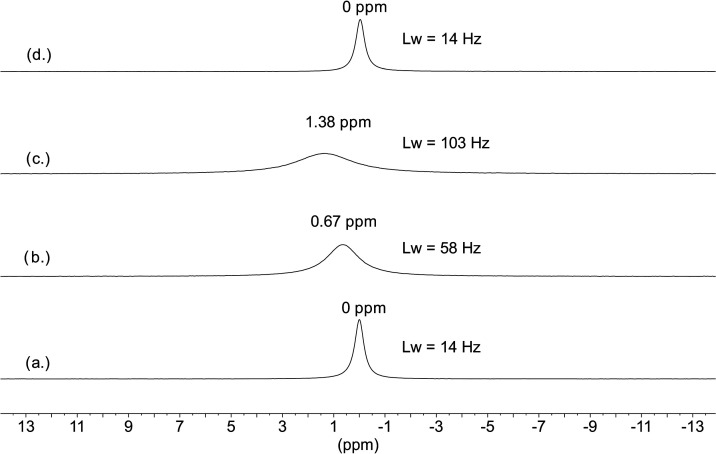
^35^Cl NMR spectra measured in aqueous solutions of (a) 5 M NaCl, (b) 5 M NaCl + 20 mM UO_2_^2+^, (c) 5 M NaCl + 20 mM UO_2_^2+^ + 20 mM H_2_O_2_, and (d) 5 M NaCl + 20 mM UO_2_^2+^ + 20 mM H_2_O_2_ + 200 mM NaHCO_3_.

The complex formation in the ternary uranyl–peroxide–chloride system was followed first by running ^35^Cl NMR experiments. Only one solution containing 5 M NaCl was used, and its composition was changed for all other NMR experiments as detailed below. The spectra are shown in Figure 8. The 5 M NaCl sample was measured first, and a relatively sharp (14 Hz) ^35^Cl signal was observed in the spectrum (Figure 8a). The chemical shift of this signal was set to 0 ppm and used as an external reference for all other spectra. After adding 20 mM uranyl to the sample, still only one signal can be observed, but with a much broader linewidth (58 Hz) and increased chemical shift at 0.67 ppm (Figure 8b). These changes in the spectral parameters clearly indicate the formation of uranyl–chloro complexes and from dynamic NMR point of view can be explained by the formation of new exchange sites for chloride. The observation of only one broad signal indicates that the exchange between the coordinated and free chloride is fast on the NMR time scale. The addition of 20 mM H_2_O_2_ to the sample increased the shift of the signal to 1.38 ppm and the linewidth to 103 Hz (Figure 8c) due to the formation of additional exchanging site(s) for chloride by coordination of peroxide to the uranyl center in the uranyl–chloro complex(es). Since the individual chemical shifts and the relative populations of the exchanging complexes are not known, the spectra serve only qualitative kinetic information in the system. Independently of the number and stoichiometry of the complexes formed, it can be stated that the coordinated chloride is kinetically labile, and it is in fast exchange with the free chloride ion.

In aqueous solutions, carbonate ion forms one of the thermodynamically most stable complexes with uranyl, and at higher carbonate concentrations, the formation of UO_2_(CO_3_)_3_^4–^ is dominating.^12,61^ We expected that carbonate would replace the weakly coordinated chlorides in the complexes formed; therefore, solid sodium bicarbonate was added to the test solution resulting in 200 mM total carbonate concentration (10 times excess relative to the uranyl concentration) and the ^35^Cl spectrum was measured again. As can be seen in Figure 8d, the signal is identical to the signal observed for free chloride in the 5 M NaCl sample (Figure 8a), indicating the presence of only free chloride ions in the solution. Hence, this observation proves unambiguously that the carbonate ion replaced the coordinated chlorides in the thermodynamically less stable uranyl–chloro and uranyl–peroxo–chloro complexes. This experiment is in accordance with our expectations and serves as indirect evidence for chloride coordination in the system.

#### ^17^O NMR

##### Uranyl–Peroxide–Chloride System

The uranyl ion, UO_2_^2+^, has two oxygen ligands, so-called “yl”-oxygens, that are chemically inert; however, by photochemical activation in ^17^O-enriched water, they can be replaced by ^17^O-isotopes. Hence, ^17^O NMR spectroscopy can also be used to follow the ligand exchange reactions.

**Figure 9:**
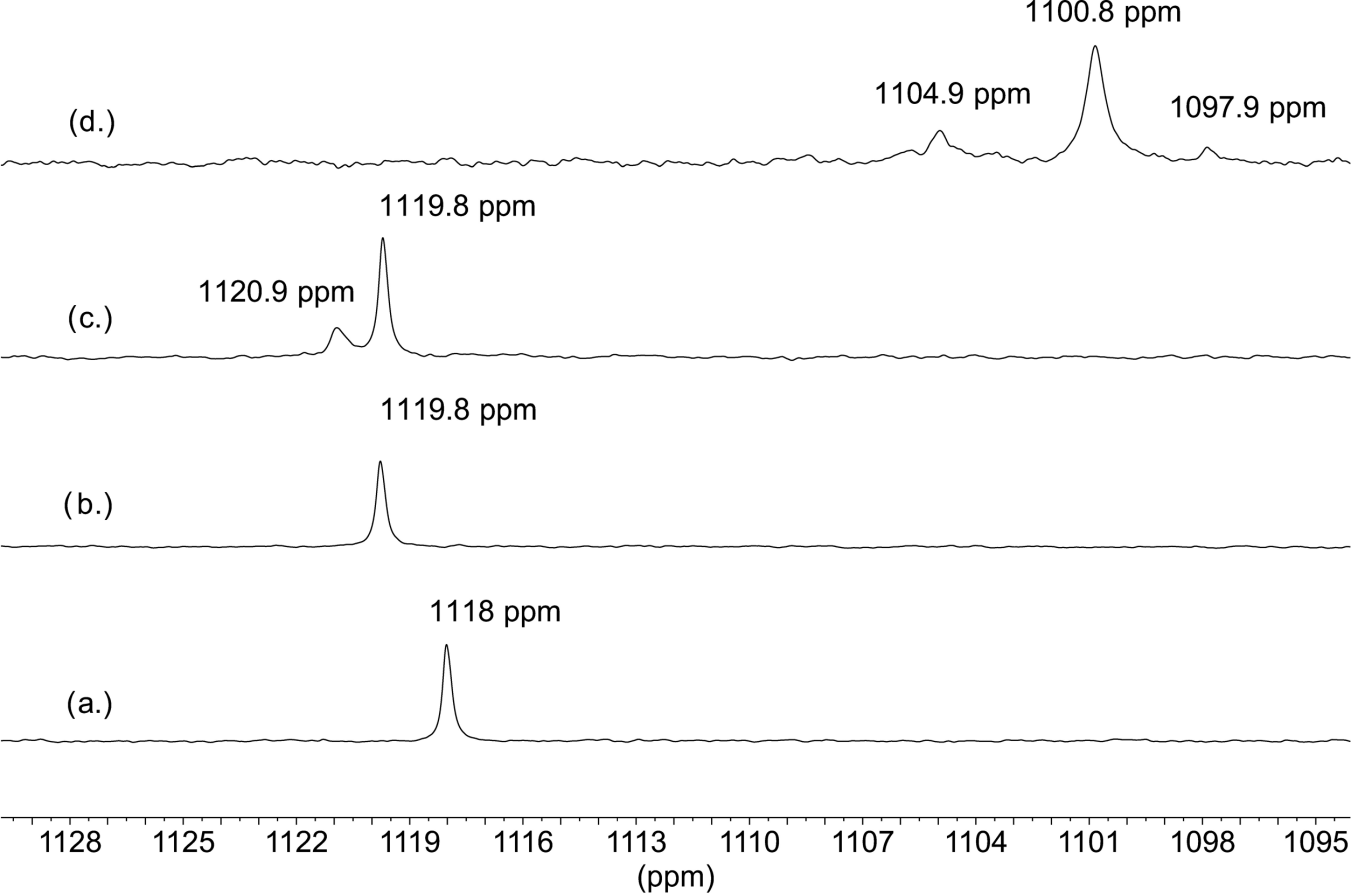
^17^O NMR spectra measured in aqueous solutions of (a) 20 mM UO_2_^2+^ in 1 M HClO_4_, (b) 5 M NaCl + 20 mM UO_2_^2+^, (c) 5 M NaCl + 20 mM UO_2_^2+^ + 20 mM H_2_O_2_, and (d) 5 M NaCl + 20 mM UO_2_^2+^ + 20 mM H_2_O_2_ + 200 mM NaHCO_3_.

First, the ^17^O NMR signal of a reference sample containing only uranyl–aquo complex, UO_2_(H_2_O)_5_^2+^ was measured. The sample contained 20 mM ^17^O-enriched uranyl in 1 M HClO_4_ to avoid the formation of uranyl–hydroxo complexes, which can contribute to the loss of ^17^O enrichment. A relatively narrow signal (14 Hz) was observed at 1118 ppm, as shown in Figure 9a. Then, similarly to the setup for the ^35^Cl NMR experiments, the ^17^O NMR spectra were recorded in 5 M NaCl using the same test solution by adding uranyl first, followed by H_2_O_2_ addition next. The ^17^O signal from the sample containing 20 mM enriched uranyl in 5 M NaCl (Figure 9b) showed the same linewidth (15 Hz) but appeared at a higher chemical shift at 1119.8 ppm relative to the signal of the uranyl aquo-ion (Figure 9a). In accordance with the observations in the corresponding ^35^Cl NMR spectra, the formation of uranyl–chloro complex(es) could result in an increase in the shift. (The same increase of the chemical shift was observed in 5 M NaBr solution as discussed below.) The addition of 20 mM H_2_O_2_ resulted in the appearance of a new signal at 1120.9 ppm (with 23 Hz linewidth), besides the signal at 1119.8 ppm (Figure 9c). The appearance of this signal, in accordance with the ^35^Cl observations, is a clear evidence of the formation of the ternary uranyl–peroxo–chloro complex. The formation of binary uranyl–peroxo complexes (without chloride coordination) can be excluded since their ^17^O chemical shifts are in the range of 1070–1080 ppm (Figure 2 in ref (31)). The appearance of individual signals for the complex(es) formed by peroxide coordination, and for the other species indicates a slow exchange between them on the ^17^O NMR time scale. It can be concluded that the coordinated peroxide is kinetically inert and not involved in the ligand exchange reactions. One should note that a fast chloride exchange between the complexes and/or with the free chloride ion in this system does not affect the chemical shift of ^17^O NMR signals. Finally, to confirm the chloride coordination in the complexes formed, similarly to the ^35^Cl NMR experiment detailed above, solid sodium bicarbonate was added to the sample resulting in 200 mM total carbonate concentration, and the ^17^O NMR spectrum was measured again. A dramatic change was observed in the spectrum (Figure 9d), CO_3_^2–^ replaced the coordinated chlorides, and signals only for the thermodynamically most stable binary carbonate, UO_2_(CO_3_)_3_^4–^ (at 1100.8 ppm), and ternary uranyl–peroxo–carbonate complexes, (UO_2_)_2_O_2_(CO_3_)_4_^6–^ (at 1104.9 ppm) and UO_2_O_2_(CO_3_)_2_^4–^ (at 1097.9 ppm), can be observed as shown in Figure 9. These complexes have been identified earlier in a detailed ^13^C and ^17^O NMR study with slightly different chemical shifts due to the differences in the experimental conditions (Figures 3 and S5 in ref (31)).

##### Uranyl–Peroxide–Bromide System

**Figure 10:**
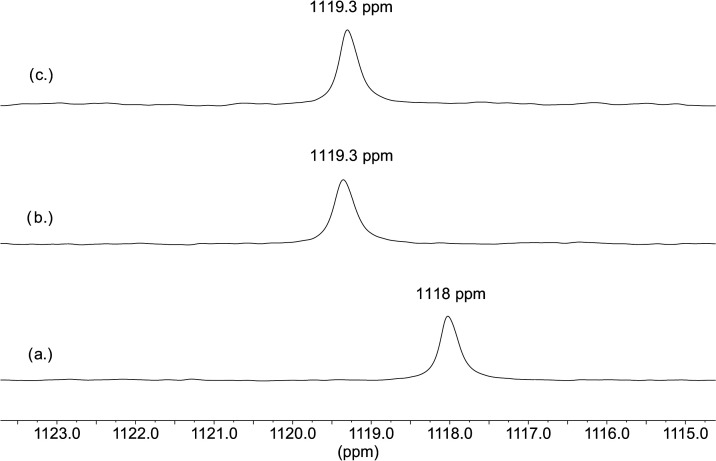
^17^O NMR spectra measured in aqueous solutions of (a) 20 mM UO_2_^2+^ in 1 M HClO_4_, (b) 5 M NaBr + 20 mM UO_2_^2+^, and (c) 5 M NaBr + 20 mM UO_2_^2+^ + 20 mM H_2_O_2_.

The ^17^O NMR spectra were recorded analogously using one test solution in 5 M NaBr by adding uranyl first and then H_2_O_2_. The ^17^O uranyl signal appeared at 1119.3 ppm in 5 M NaBr, slightly lower than that observed for the corresponding sample in 5 M NaCl but still higher than the shift of the uranyl–aquo complex (Figure 10a,b) and can be explained by the formation of uranyl–bromo complex. After the addition of H_2_O_2_, the solution starts to be cloudy after ∼10 min indicating the formation of studtite and only one signal with the same shift can be observed (Figure 10c). This could be rationalized by the ternary uranyl–peroxo–bromo complex being considerably weaker than the corresponding chloro complex. The weak bromo complex is transformed into thermodynamically more stable studtite on a time scale shorter than that required for recording the NMR spectrum.

To the best of our knowledge, the spectroscopical results presented above constitute the first experimental observations of the formation of ternary uranyl–peroxo–chloro and uranyl–peroxo–bromo complexes.

### Stoichiometry and Stability of the Ternary Complexes

Based on the Raman results, we propose the stoichiometry of the observed ternary complexes to be UO_2_(O_2_)(Cl)(H_2_O)_2_^–^ and UO_2_(O_2_)(Br)(H_2_O)_2_^–^. The uranyl chloro and bromo complexes identified by Raman and IR spectra are UO_2_Cl^+^ and UO_2_Br^+^ according to the literature.^10^ After adding H_2_O_2_ to the solution, the ^35^Cl NMR indicates that the Cl is still in the complex and we therefore conclude that the ternary complex also contains one chloride. In addition, the Raman spectrum of studtite shows one peak at 821 cm^–1^. The ratio between UO_2_^2+^ and O_2_^2–^ in studtite is 1:1, and given the fact that the Raman peaks of the uranyl–peroxo–halo complexes are found around 830 cm^–1^, which is close to that of studtite, we conclude that the ratio between UO_2_^2+^ and O_2_^2–^ in the complexes is also 1:1. DFT calculations performed by Odoh and Schreckenbach^33^ to simulate the Raman peak of the symmetric stretch of UO_2_^2+^ coordinated to different numbers of O_2_^2–^ show that the predicted symmetric stretch of UO_2_^2+^ in UO_2_(O_2_)(H_2_O)_4_ and UO_2_(O_2_)_2_(H_2_O)_2_ should be at 789 and 704 cm^–1^, respectively. This indicates a significant redshift of UO_2_^2+^ vibration frequency upon coordination to additional O_2_^2–^. The relatively high wavenumbers of the UO_2_^2+^ symmetric stretch of the uranyl–peroxo–halo complexes imply that the complex only contains one O_2_^2–^. In addition, the unsaturated UO_2_(O_2_)(Cl)^−^ or UO_2_(O_2_)(Br)^−^ needs to bind two H_2_O molecules to achieve equatorial coordination numbers of 5.^64^ Based on Raman measurements, the estimated stability constants for (UO_2_)(O_2_)(Cl)(H_2_O)_2_^–^ and (UO_2_)(O_2_)(Br)(H_2_O)_2_^–^ are 0.17 and 0.04, respectively, at ionic strength ≈5 mol/L (based on eqs 1 and 2, X = Cl^–^/Br^–^).
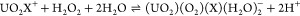
1
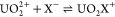
2

The details of the estimations are given in the Supporting Information. Interestingly, speciation calculations based on the estimated stability constants (Figures S18 and S19) show that the ternary complexes dominate the speciation at lower UO_2_^2+^ and H_2_O_2_ concentrations (0.2 mM). Moreover, speciation calculations performed as a function of pH show that the ternary complexes are the dominating species in 1 M Cl^–^/Br^–^ solutions at pH higher than 3 (Figures S22 and S23).

The confirmation of the existence of the uranyl–peroxo–chloro and uranyl–peroxo–bromo complexes completes the picture of the recently performed study on H_2_O_2_-induced oxidative dissolution of UO_2_ in saline aqueous solutions.^19^ The ternary peroxo complexes act as unreactive sinks for H_2_O_2_ in the system while the fraction of free H_2_O_2_ displays reactivity toward the UO_2_ surface. The technique used for quantitative analysis of H_2_O_2_ is not specific for free H_2_O_2_ and instead, the total peroxide concentration is measured. This explains why, under certain conditions, the consumption of H_2_O_2_ appears to slow down considerably once the concentration of UO_2_^2+^ has increased.

## Conclusions

This work shows that ionic strength significantly influences the rate of studtite formation. At *I* ≈ 1 mol/L, the formation of studtite in solutions containing 0.2 mM UO_2_^2+^ and 0.2 mM H_2_O_2_ can be completely suppressed for at least 24 h. Dissolution experiments also show that the rate of studtite dissolution increases with increasing ionic strength and that Cl^–^ and Br^–^ are more effective than ClO_4_^–^. In the presence of uranyl, H_2_O_2_ can be consumed in Br^–^ solutions in two processes. One is catalytic decomposition, and the other is the formation of studtite. The catalytic decomposition was shown to involve a more reactive intermediate as evidenced by the oxidation of indigo carmine. The existence of ternary uranyl–peroxo–chloro and uranyl–peroxo–bromo complexes has been confirmed by Raman and IR spectroscopy. Raman spectra show that the symmetric stretch (ν_1_) of UO_2_^2+^ in uranyl–peroxo–chloro and uranyl–peroxo–bromo complexes are at 831 and 829 cm^–1^, respectively. The estimated stability constants for (UO_2_)(O_2_)(Cl)(H_2_O)_2_^–^ and (UO_2_)(O_2_)(Br)(H_2_O)_2_^–^ are 0.17 and 0.04, respectively, at *I* ≈ 5 mol/L. Speciation calculations based on these stability constants show that the ternary complexes dominate the speciation at lower concentrations of UO_2_^2+^ and H_2_O_2_ at pH ≥ 3. IR spectra show that the asymmetric stretch (ν_3_) of UO_2_^2+^ in uranyl–peroxo–chloro and uranyl–peroxo–bromo complexes are at 925 and 927 cm^–1^, respectively. In addition, the ^35^Cl and ^17^O NMR spectra show that the coordinated chlorides are kinetically labile, and they are in fast exchange with the free chloride, while the exchange reactions between the coordinated and free peroxides are slow on the ^17^O NMR time scale that makes possible to observe separate signal for the uranyl–peroxo–chloro complex.

## Supporting Information Available

Speciation calculations for uranyl chloride and uranyl bromide system; results from time-resolved studies of [UO_2_^2+^] and [H_2_O_2_] in aqueous solutions; pH measurements for different samples; pH effect on H_2_O_2_ decomposition; summary of peaks measured in Raman and IR spectra; stability constants calculation; sample photographs at different times; XRD for precipitates formed in aqueous solutions; and speciation calculations based on the estimated stability constants for the characterized ternary complexes (PDF)
