# Supplementary material for: Stability of Studtite in Saline Solution: Identification of Uranyl–Peroxo–Halo Complex
Source: Inorg Chem. 2022 May 24;61(22):8455–66. doi: 10.1021/acs.inorgchem.2c00233 (PMC9175179; doi:10.1021/acs.inorgchem.2c00233)
Supplement: Supplementary file 1 — ic2c00233_si_001.pdf [file ic2c00233_si_001.pdf]

Supporting Information

Stability of studtite in saline solution. The identification of uranyl-peroxo-halo complex.

Junyi Li,* Zoltán Szabó and Mats Jonsson

Table of Contents

Results and discussions……2
Speciation calculations for uranyl chloride and uranyl bromide system……2
Equilibrium constants of various species used in speciation simulation……4
Results from time-resolved studies of [UO_2^{2+}] and [H_2O_2] in aqueous solutions……5
pH measurements for different samples……7
pH effect on H_2O_2 decomposition……7
Summary of peaks measured in Raman and IR spectra……8
Stability constants calculation……9
Sample photographs at different time……10
XRD for precipitates formed in aqueous solutions……12
Speciation calculations based on the estimated stability constants for the characterized ternary complexes……13
References……16

Results and Discussion

Speciation calculations for uranyl chloride and uranyl bromide systems

0.2 mM and 20 mM UO_2^{2+} in 0-5 M Cl^- system

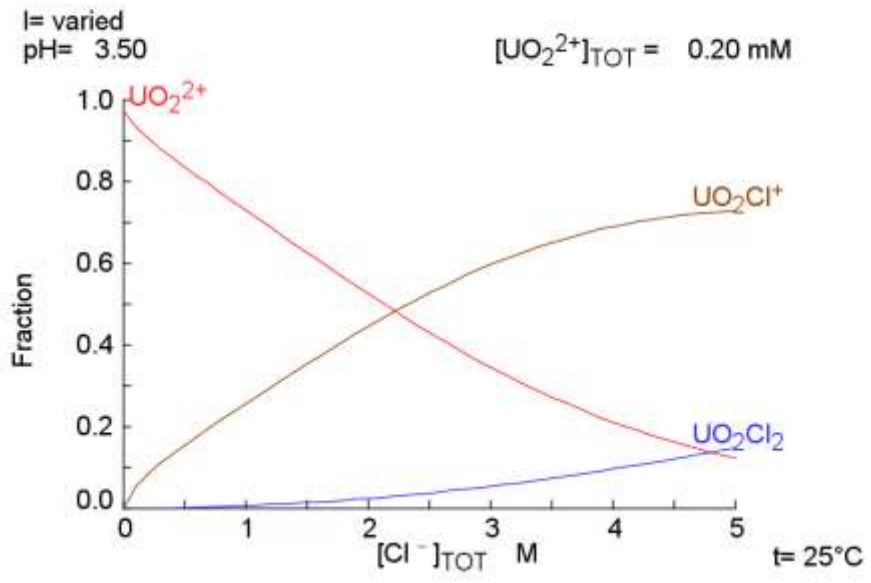

Figure S1. Speciation simulation for 0.2 mM UO_2^{2+} in chloride system.[1] The ionic strength was calculated using SIT (Specific ion Interaction Theory) method.

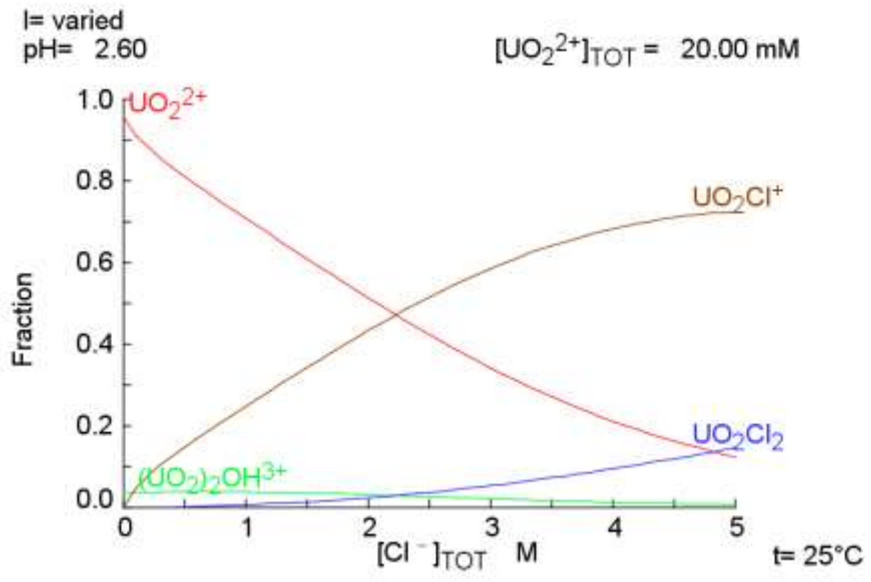

Figure S2. Speciation simulation for 20 mM UO_2^{2+} in chloride system.[1] The ionic strength was calculated using SIT method.

0.2 mM and 20 mM UO_2^{2+} in 0-5 M Br^- system

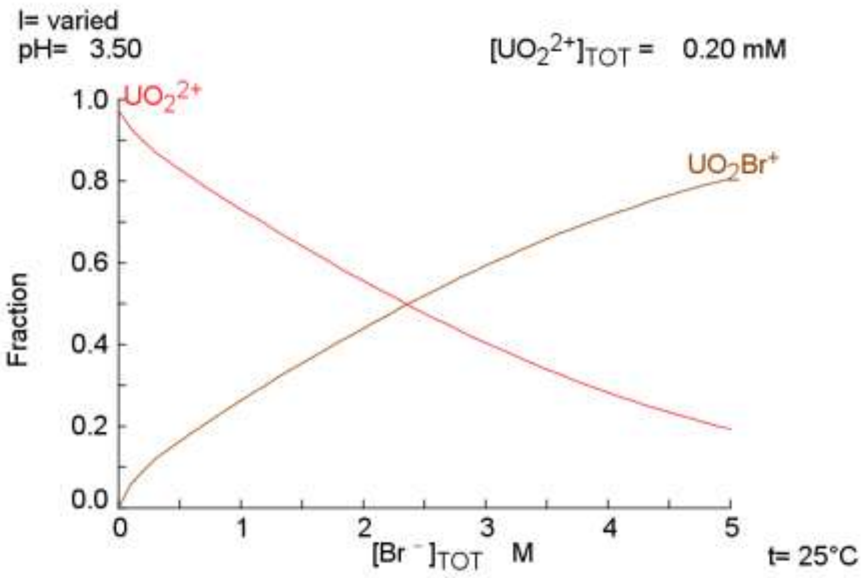

Figure S3. Speciation simulation for 0.2 mM UO_2^{2+} in bromide system.[1] The ionic strength was calculated using SIT method.

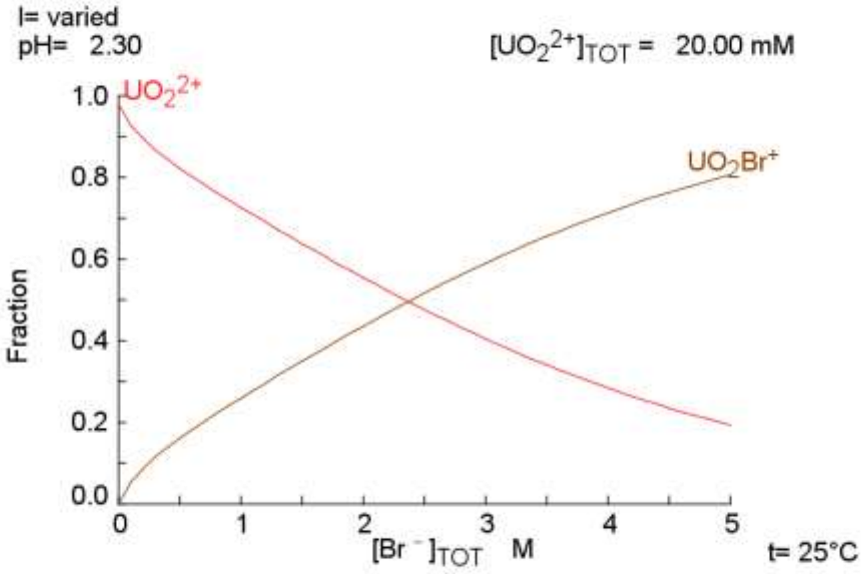

Figure S4. Speciation simulation for 20 mM UO_2^{2+} in bromide system.[1] The ionic strength was calculated using SIT method.

The speciation simulation performed in figure S1-S4 was done using Medusa program[2], and the equilibrium constants of species are shown in table S1.

Table S1. equilibrium constants of various species used in speciation simulation

species	equilibrium constants[1]
$(UO_2)_2(OH)_2^{2+}$	-5.62
$(UO_2)_2OH^{3+}$	-2.7
$(UO_2)_3(OH)_4^{2+}$	-11.9
$(UO_2)_3(OH)^{5+}$	-15.55
$(UO_2)_3(OH)_7^{-}$	-32.2
$(UO2)_4(OH)_7^{+}$	-21.9
$UO_2(OH)_2$	-12.15
$UO_2(OH)_3^{-}$	-20.25
$UO_2(OH)_4^{2-}$	-32.4
UO_2Cl^{+}	0.17
UO_2Cl_2	-1.1
UO_2Br^{+}	0.22
UO_2OH^{+}	-5.25
$UO_2(OH)_2.H_2O$	-4.81

Results from time-resolved studies of $[UO_2^{2+}]$ and $[H_2O_2]$ in aqueous solutions

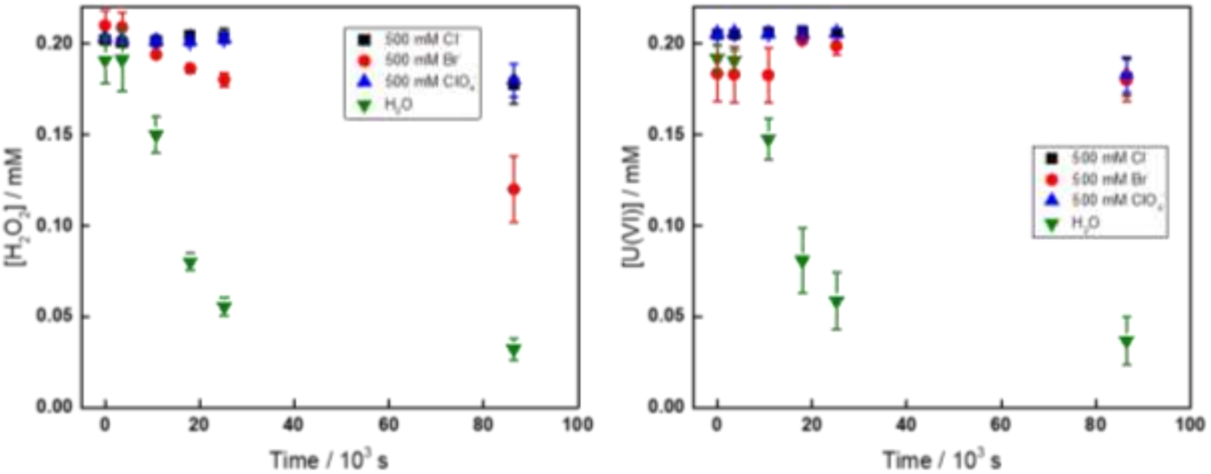

Figure S5. The concentrations of H_2O_2 (left) and UO_2^{2+}(right) in 500 mM Cl^-, Br^-, ClO_4^- solutions and H_2O as a function of time.

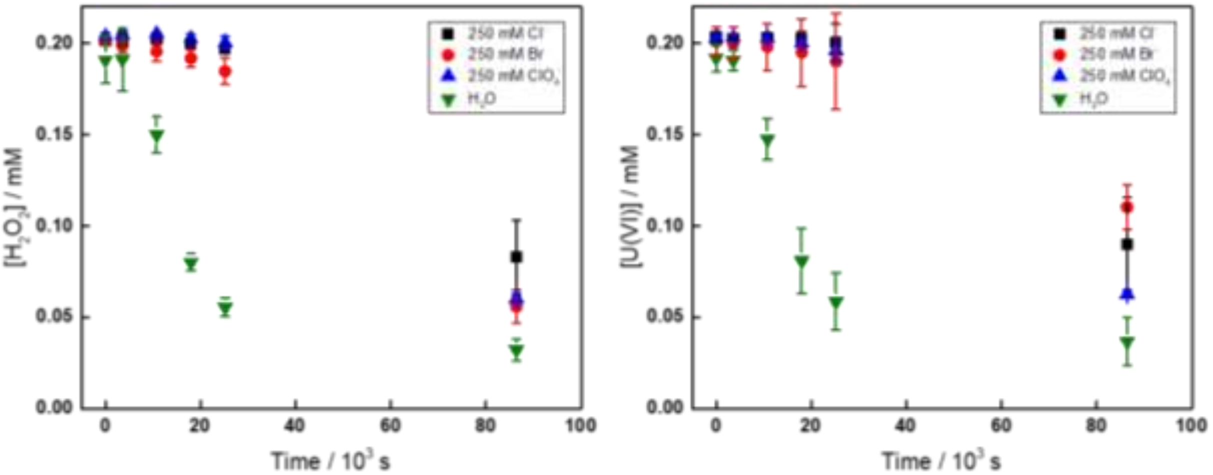

Figure S6. The concentrations of H_2O_2 (left) and UO_2^{2+}(right) in 250 mM Cl^-, Br^-, ClO_4^- solutions and H_2O as a function of time.

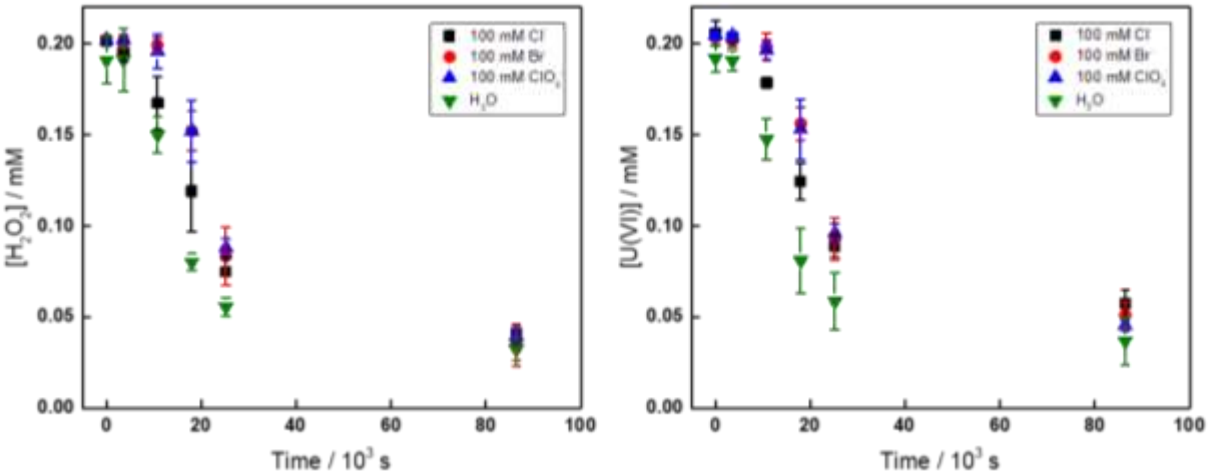

Figure S7. The concentrations of H_2O_2 (left) and UO_2^{2+}(right) in 100 mM Cl^-, Br^-, ClO_4^- solutions and H_2O as a function of time.

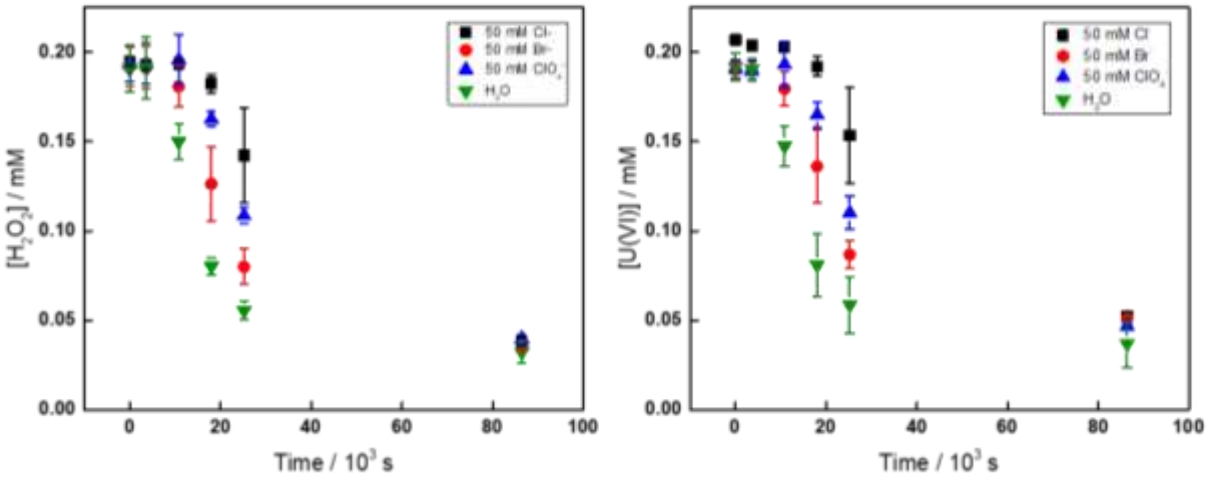

Figure S8. The concentrations of H_2O_2 (left) and UO_2^{2+}(right) in 50 mM Cl^-, Br^-, ClO_4^- solutions and H_2O as a function of time.

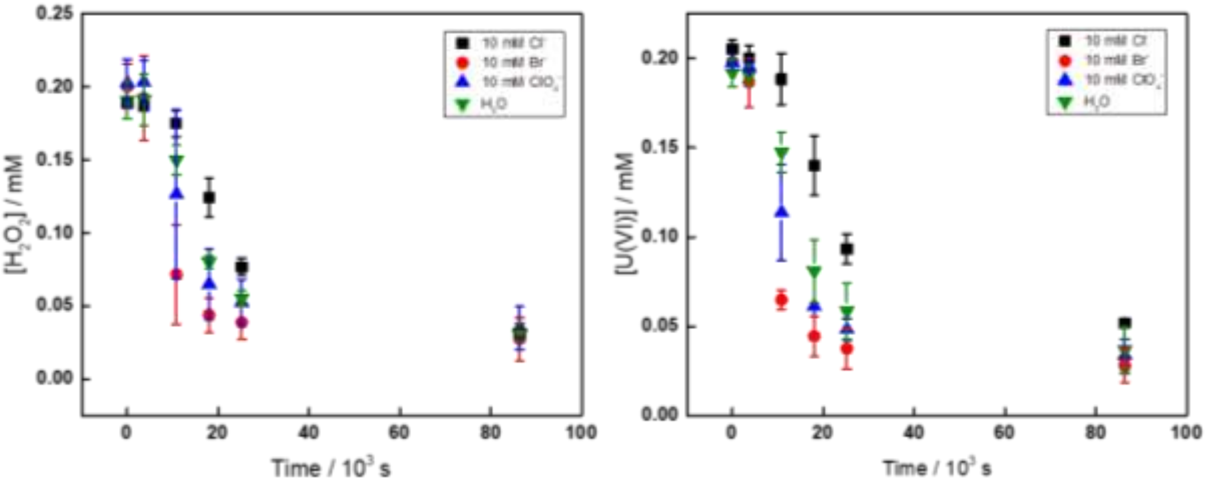

Figure S9. The concentrations of H_2O_2 (left) and UO_2^{2+}(right) in 10 mM Cl^-, Br^-, ClO_4^- solutions and H_2O as a function of time.

pH measurements

Table S2. pH valuse for the samples used in different experiments.

Sample composition	pH after sample preparation	pH in the end of the experiment
Time-resolved studies of [UO_2^{2+}] and [H_2O_2] in aqueous solutions		
0.2 mM UO_2^{2+} and 0.2 mM H_2O_2 in H_2O	3.69	3.63
0.2 mM UO_2^{2+} and 0.2 mM H_2O_2 in 1 M Cl^-	3.67	3.73
0.2 mM UO_2^{2+} and 0.2 mM H_2O_2 in 1 M Br^-	3.64	4.35
0.2 mM UO_2^{2+} and 0.2 mM H_2O_2 in 1 M ClO_4^-	3.63	3.58
0.2 mM UO_2^{2+} and 0.2 mM H_2O_2 in 500 mM Cl^-	3.68	3.77
0.2 mM UO_2^{2+} and 0.2 mM H_2O_2 in 500 mM Br^-	3.69	3.85
0.2 mM UO_2^{2+} and 0.2 mM H_2O_2 in 500 mM ClO_4^-	3.64	3.59
0.2 mM UO_2^{2+} and 0.2 mM H_2O_2 in 250 mM Cl^-	3.81	3.82
0.2 mM UO_2^{2+} and 0.2 mM H_2O_2 in 250 mM Br^-	3.78	3.83
0.2 mM UO_2^{2+} and 0.2 mM H_2O_2 in 250 mM ClO_4^-	3.71	3.59
0.2 mM UO_2^{2+} and 0.2 mM H_2O_2 in 100 mM Cl^-	3.76	3.67
0.2 mM UO_2^{2+} and 0.2 mM H_2O_2 in 100 mM Br^-	3.78	3.64
0.2 mM UO_2^{2+} and 0.2 mM H_2O_2 in 100 mM ClO_4^-	3.79	3.60
0.2 mM UO_2^{2+} and 0.2 mM H_2O_2 in 50 mM Cl^-	3.87	3.70
0.2 mM UO_2^{2+} and 0.2 mM H_2O_2 in 50 mM Br^-	3.79	3.70
0.2 mM UO_2^{2+} and 0.2 mM H_2O_2 in 50 mM ClO_4^-	3.80	3.71
0.2 mM UO_2^{2+} and 0.2 mM H_2O_2 in 10 mM Cl^-	3.74(at 7 hours)	-
0.2 mM UO_2^{2+} and 0.2 mM H_2O_2 in 10 mM Br^-	3.57(at 7 hours)	-
0.2 mM UO_2^{2+} and 0.2 mM H_2O_2 in 10 mM ClO_4^-	3.64(at 7 hours)	-
Studtite stability experiment		
Studtite dissolution in 1 M Cl^-	5.56	6.74
Studtite dissolution in 5 M Cl-	6.35	5.75
Studtite dissolution in 1 M Br^-	7.28	7.01
Studtite dissolution in 5 M Br^-	5.50	5.05
Studtite dissolution in 1 M ClO_4^-	4.90	5.62
Studtite dissolution in 5 M ClO_4^-	4.62	4.83
**Spectroscopy**		
20 mM UO_2^{2+} in H_2O	3.09	-
20 mM UO_2^{2+} and 20 mM H_2O_2 in H_2O	1.75	-
20 mM UO_2^{2+} in 5 M Cl^-	2.61	-
20 mM UO_2^{2+} and 20 mM H_2O_2 in 5 M Cl^-	1.17	-
20 mM UO_2^{2+} in 5 M Br^-	2.26	-
20 mM UO_2^{2+} and 20 mM H_2O_2 in 5 M Br^-	1.04	-
20 mM UO_2^{2+} in 1 M $HClO_4$	-	-

Table S3. pH effect on H_2O_2 decomposition

Time (h)	0.2 mM U(VI) and H_2O_2 in 1 M NaBr (pH=4.04)	HCl acidified 1 M NaBr and H_2O_2 (pH=4.04)	H_2O_2 and 1 M NaBr (pH=5.80)
	H_2O_2 concentration / mM	H_2O_2 concentration / mM	H_2O_2 concentration / mM
0	0.173	0.176	0.174
3	0.170	0.175	0.173
6	0.158	0.176	0.175
30	0.085	0.172	0.170
48	0.018	0.161	0.161

Raman peaks

Table S4. summary of peaks measured in Raman spectra

Sample composition	Peak position (cm^{-1})	Peak indication	reference
20 mM U(VI) in H_2O	871	$UO_2(H_2O)_5^{2+}$	[3, 4, 5, 6]
20 mM H_2O_2 and 20 mM U(VI) in H_2O	821	$UO_2O_2.4H_2O$ (studtite)	[7, 8, 9, 10]
	735	amorphous uranyl peroxide	[9]
	870	O-O stretch in studtite	[11, 10, 9, 12]
20 mM U(VI) in 5M NaCl	865	UO_2Cl^+	[3]
20 mM U(VI) and 20 mM H_2O_2 in 5M NaCl	865	UO_2Cl^+	[3]
	831	$UO_2O_2Cl^-$	present work
20 mM U(VI) and 20 mM H_2O_2 in 5M NaBr	871	UO_2Br^+	[3]
	830	$UO_2O_2Br^-$	present work
20 mM U(VI) in 5M NaBr	871	UO_2Br^+	[3]
20 mM H_2O_2 in H_2O	877	O-O stretch	[13, 14, 15]

..

IR peaks

Table S5. summary of peaks measured in IR spectra

Sample composition	Peak position (cm^{-1})	Peak indication	reference
20 mM U(VI) in H_2O	962	$UO_2(H_2O)_5^{2+}$	[6, 16]
20 mM H_2O_2 and 20 mM U(VI) in H_2O	905	UO2O2.4H2O	[9, 10, 17]
20 mM U(VI) in 5M NaCl	948	UO_2Cl^+	Present work
60 mM U(VI) and 40 mM H_2O_2 in 5M NaCl	948	UO_2Cl^+	Present work
	925	$UO_2O_2Cl^-$	Present work
60 mM U(VI) and 40 mM H_2O_2 in 5M NaBr	960	UO_2Br^+	Present work
	927	$UO_2O_2Br^-$	Present work
20 mM U(VI) in 5M NaBr	960	UO_2Br^+	Present work

Stability constants calculation

For Cl^- system

$$UO_2Cl^+ + H_2O_2 + 2H_2O \rightleftharpoons (UO_2)(O_2)(Cl)(H_2O)_2^- + 2H^+ \qquad (1)$$

$$K_1 = \frac{[(UO_2)(O_2)(Cl)(H_2O)_2^-]\cdot[H^+]^2}{[UO_2Cl^+]_{final}\cdot[H_2O_2]} = 0.114\ M$$

$[UO_2Cl^+]_{final} = [UO_2Cl^+]_{initial} - [(UO_2)(O_2)(Cl)(H_2O)_2^-] = 20\ mM - 5.36\ mM = 1.464*10^{-2}\ M$

$[H^+] = 6.76 *10^{-2}\ M$ (pH = 1.17)

$[H_2O_2] = [H_2O_2]_{total} - [(UO_2)(O_2)(Cl)(H_2O)_2^-] = 20\ mM - 5.36\ mM = 1.464*10^{-2}\ M$

$$UO_2^{2+} + Cl^- \rightleftharpoons UO_2Cl^+ \qquad (2)$$

$K_2 = 1.5\ M^{-1}$ (Reference a)

$K = K_1 K_2 = 0.17$ (Ionic strength ≈ 5 M/L)

Note: All the U(VI) was estimated in the form of UO_2Cl^+ initially,eg., $[UO_2Cl^+]$ is 20 mM initially
$[(UO_2)(O_2)(Cl)(H_2O)_2^-]$ was estimated by the relative change in the $[UO_2Cl]^+$ peak upon addition of H_2O_2.

For Br^- system

$$UO_2Br^+ + H_2O_2 + 2H_2O \rightleftharpoons (UO_2)(O_2)(Br)(H_2O)_2^- + 2H^+ \qquad (1)$$

$$K_1 = \frac{[(UO_2)(O_2)(Br)(H_2O)_2^-]\cdot[H^+]^2}{[UO_2Br^+]_{final}\cdot[H_2O_2]} = 0.1\ M$$

$[UO_2Br^+]_{final} = [UO_2Br^+]_{initial} - [(UO_2)(O_2)(Br)(H_2O)_2^-] = 20\ mM - 3.333\ mM = 1.667*10^{-2}\ M$

$[H^+] = 9.12 *10^{-2}\ M$ (pH = 1.04)

$[H_2O_2] = [H_2O_2]_{total} - [(UO_2)(O_2)(Br)(H_2O)_2^-] = 20\ mM - 3.333\ mM = 1.667*10^{-2}\ M$

$$UO_2^{2+} + Br^- \rightleftharpoons UO_2Br^+ \qquad (2)$$

$K_2 = 0.4\ M^{-1}$ (Reference b)

$K = K_1 K_2 = 0.04$ (Ionic strength ≈ 5 M/L)

Note: All the U(VI) was estimated in the form of UO_2Br^+ initially,eg., $[UO_2Br^+]$ is 20 mM initially
$[(UO_2)(O_2)(Br)(H_2O)_2^-]$ was estimated by the relative change in the $[UO_2Br]^+$ peak upon addition of H_2O_2.

Reference a[18]; reference b[1].

Sample photographs at different time

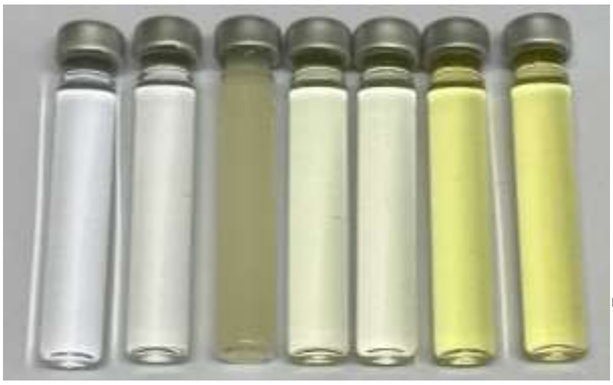

T=1 minute

Figure S10. From left to right **(1)**. H_2O **(2)**. 20 mM UO_2^{2+} in H_2O **(3)**. 20 mM UO_2^{2+} and 20 mM H_2O_2 in H_2O **(4)**. 20 mM UO_2^{2+} in 5 M NaCl **(5)**. 20 mM UO_2^{2+} in 5 M NaBr **(6)**. 20 mM UO_2^{2+} and 20 mM H_2O_2 in 5 M Cl **(7)**. 20 mM UO_2^{2+} and 20 mM H_2O_2 in 5 M Br at 1 minute after sample preparation.

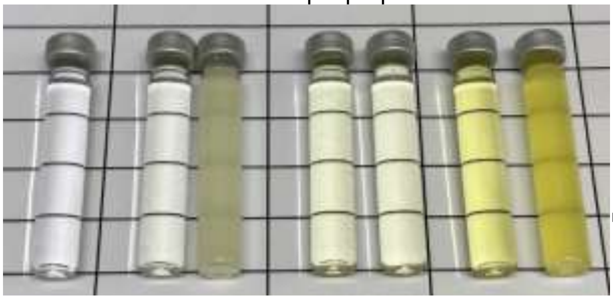

T=15 minutes

Figure S11. From left to right **(1)**. H_2O **(2)**. 20 mM UO_2^{2+} in H_2O **(3)**. 20 mM UO_2^{2+} and 20 mM H_2O_2 in H_2O **(4)**. 20 mM UO_2^{2+} in 5 M NaCl **(5)**. 20 mM UO_2^{2+} in 5 M NaBr **(6)**. 20 mM UO_2^{2+} and 20 mM H_2O_2 in 5 M Cl **(7)**. 20 mM UO_2^{2+} and 20 mM H_2O_2 in 5 M Br at 15 minutes after sample preparation.

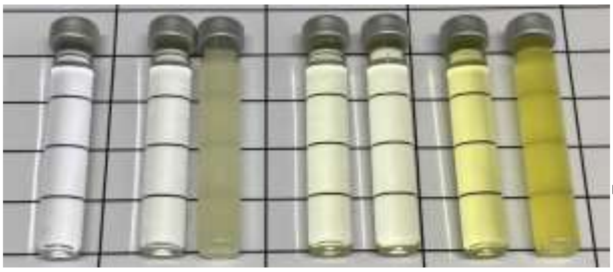

T=1 hour

Figure S12. From left to right **(1)**. H_2O **(2)**. 20 mM UO_2^{2+} in H_2O **(3)**. 20 mM UO_2^{2+} and 20 mM H_2O_2 in H_2O **(4)**. 20 mM UO_2^{2+} in 5 M NaCl **(5)**. 20 mM UO_2^{2+} in 5 M NaBr **(6)**. 20 mM UO_2^{2+} and 20 mM H_2O_2 in 5 M Cl **(7)**. 20 mM UO_2^{2+} and 20 mM H_2O_2 in 5 M Br at 1 hour**.**

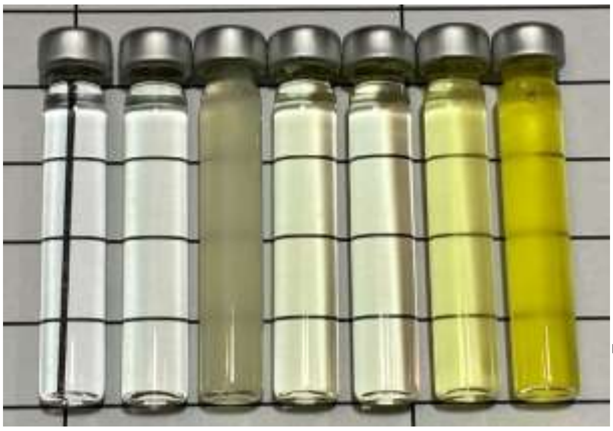

T=3 hours

Figure S13. From left to right **(1)**. H_2O **(2)**. 20 mM UO_2^{2+} in H_2O **(3)**. 20 mM UO_2^{2+} and 20 mM H_2O_2 in H_2O **(4)**. 20 mM UO_2^{2+} in 5 M NaCl **(5)**. 20 mM UO_2^{2+} in 5 M NaBr **(6)**. 20 mM UO_2^{2+} and 20 mM H_2O_2 in 5 M Cl **(7)**. 20 mM UO_2^{2+} and 20 mM H_2O_2 in 5 M Br at 3 hour.

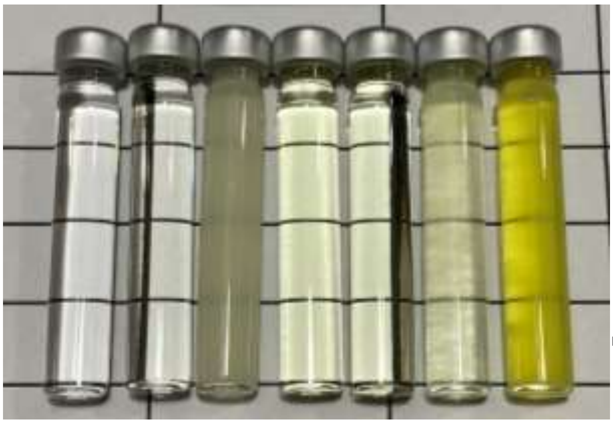

T=26 hours

Figure S14. From left to right **(1)**. H_2O **(2)**. 20 mM UO_2^{2+} in H_2O **(3)**. 20 mM UO_2^{2+} and 20 mM H_2O_2 in H_2O **(4)**. 20 mM UO_2^{2+} in 5 M NaCl **(5)**. 20 mM UO_2^{2+} in 5 M NaBr **(6)**. 20 mM UO_2^{2+} and 20 mM H_2O_2 in 5 M Cl **(7)**. 20 mM UO_2^{2+} and 20 mM H_2O_2 in 5 M Br at 26 hours.

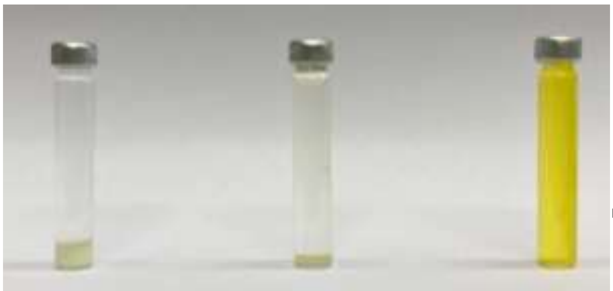

T=4 months

Figure S15. From left to right **(1)**. 20 mM UO_2^{2+} and 20 mM H_2O_2 in H_2O **(2)**. 20 mM UO_2^{2+} and 20 mM H_2O_2 in 5 M Cl^- **(3)**. 20 mM UO_2^{2+} and 20 mM H_2O_2 in 5 M Br^- after 4 months.

XRD
XRD for precipitates formed in aqueous solutions

For Cl$^-$ system

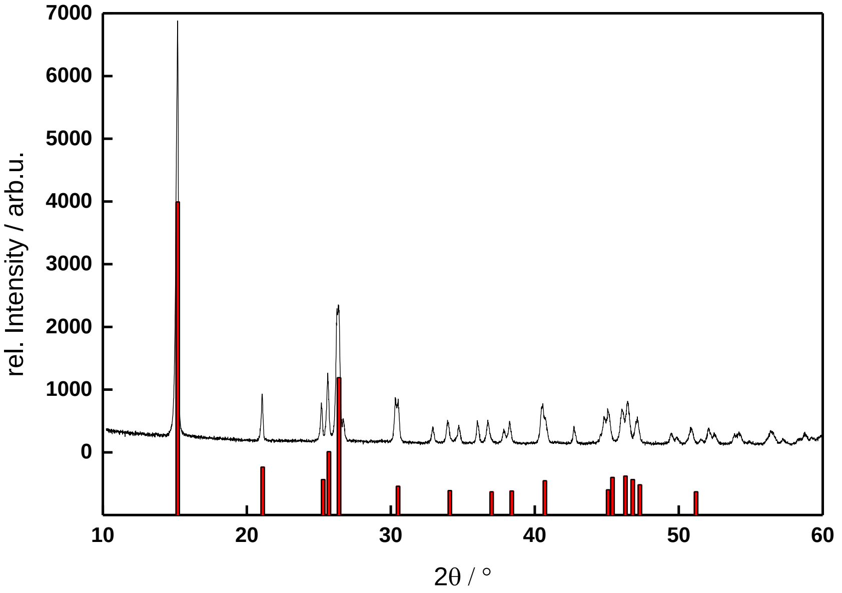

Figure S16. XRD pattern of the precipitate formed after mixing 20 mM U(VI) and 20 mM H_2O_2 in 2 M Cl$^-$ for 5 days. The red lines represent the reference diffractogram for studtite.

Br$^-$ system

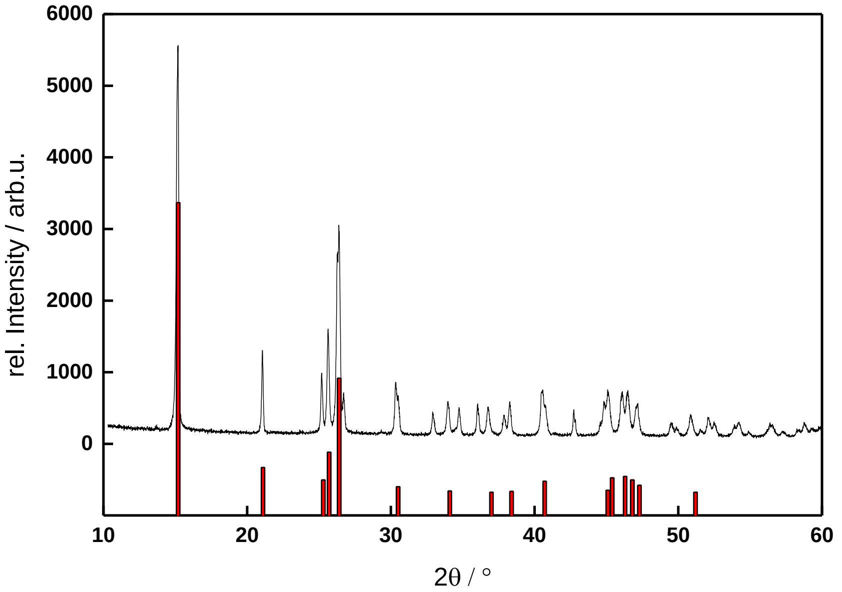

Figure S17. XRD pattern of the precipitate formed after mixing 20 mM U(VI) and 20 mM H_2O_2 in 2 M Br$^-$ for 5 days. The red lines represent the reference diffractogram for studtite.

Speciation calculations based on the estimated stability constants for the characterized ternary complexes

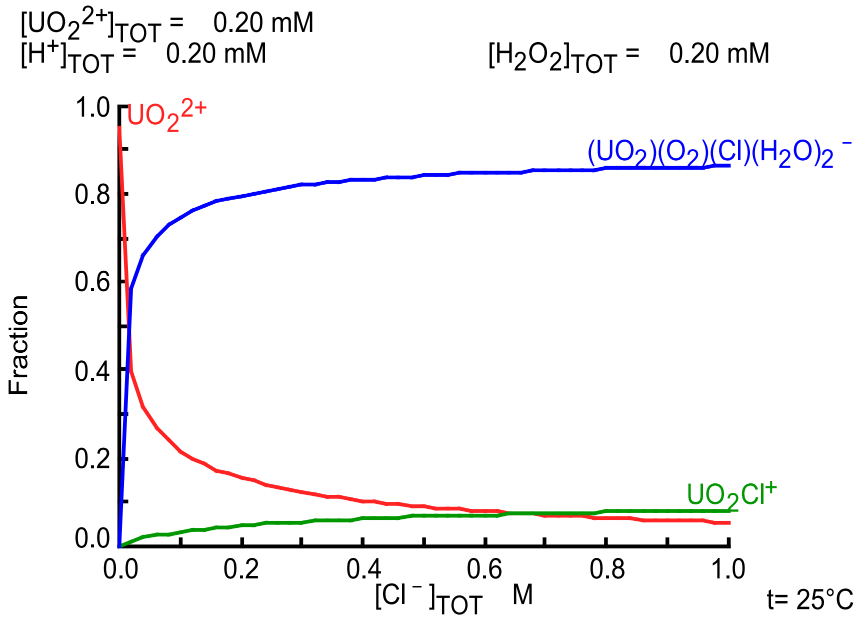

Figure S18. Speciation calculation for 0.2 mM UO_2^{2+} and 0.2 mM H_2O_2 in 0-1 M Cl^- solution based on the estimated stability constant for $(UO_2)(O_2)(Cl)(H_2O)_2^-$. The stability constants used for UO_2Cl^+ and $(UO_2)(O_2)(Cl)(H_2O)_2^-$ are valid at Ionic strength ≈ 5 mol/L.

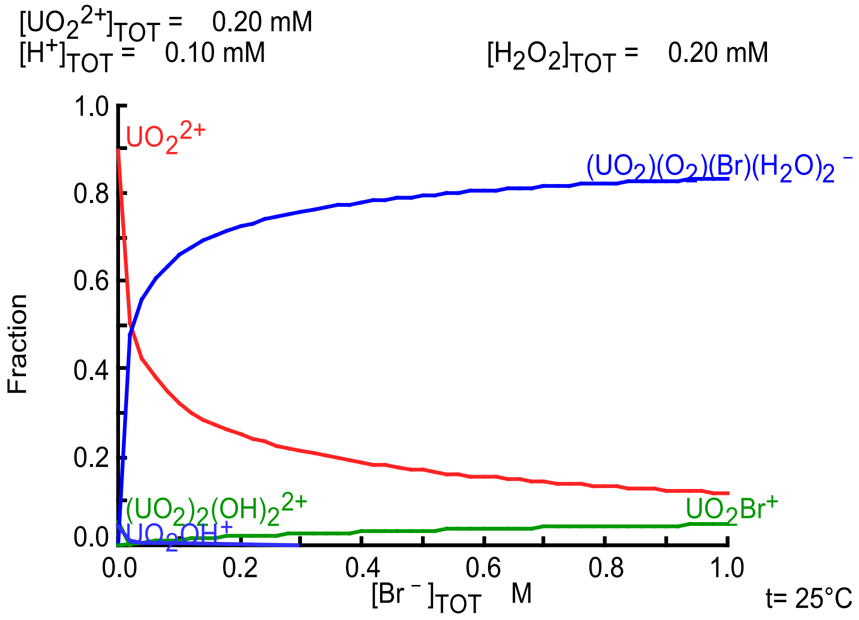

Figure S19. Speciation calculation for 0.2 mM UO_2^{2+} and 0.2 mM H_2O_2 in 0-1 M Br^- solution based on the estimated stability constant for $(UO_2)(O_2)(Br)(H_2O)_2^-$. The stability constants used for UO_2Br^+ and $(UO_2)(O_2)(Br)(H_2O)_2^-$ are valid at Ionic strength ≈ 5 mol/L.

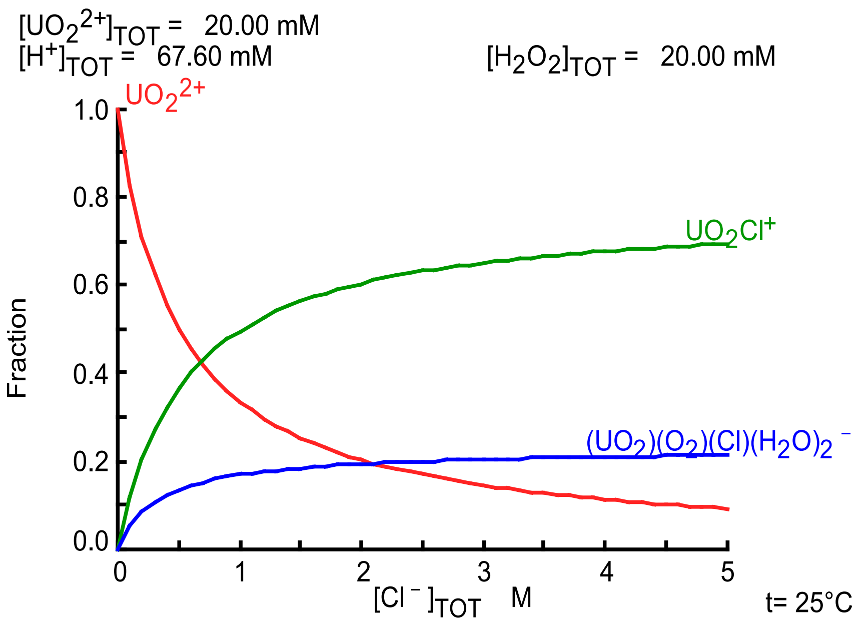

Figure S20. Speciation calculation for 20 mM UO_2^{2+} and 20 mM H_2O_2 in 0-5 M Cl^- solution based on the estimated stability constant for $(UO_2)(O_2)(Cl)(H_2O)_2^-$. The stability constants used for UO_2Br^+ and $(UO_2)(O_2)(Cl)(H_2O)_2^-$ are valid at Ionic strength ≈ 5 mol/L.

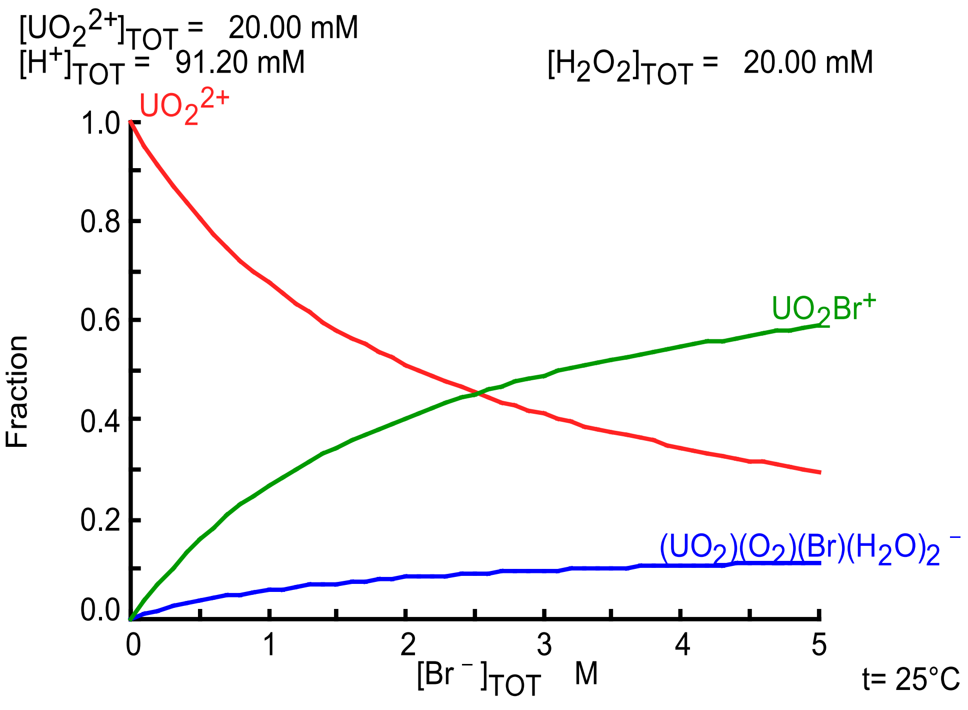

Figure S21. Speciation calculation for 20 mM UO_2^{2+} and 20 mM H_2O_2 in 0-5 M Br^- solution based on the estimated stability constant for $(UO_2)(O_2)(Br)(H_2O)_2^-$. The stability constants used for UO_2Br^+ and $(UO_2)(O_2)(Br)(H_2O)_2^-$ are valid at Ionic strength ≈ 5 mol/L.

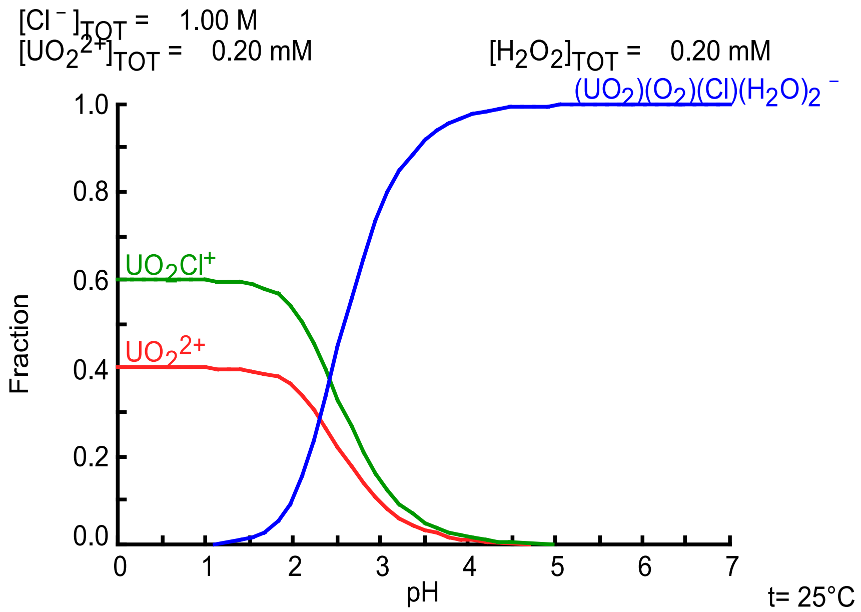

Figure S22. Speciation calculation for 0.2 mM UO_2^{2+} and 0.2 mM H_2O_2 in 1 M Cl^- solution as a function of pH based on the estimated stability constant for $(UO_2)(O_2)(Cl)(H_2O)_2^-$. The stability constants used for UO_2Br^+ and $(UO_2)(O_2)(Cl)(H_2O)_2^-$ are valid at Ionic strength ≈ 5 mol/L.

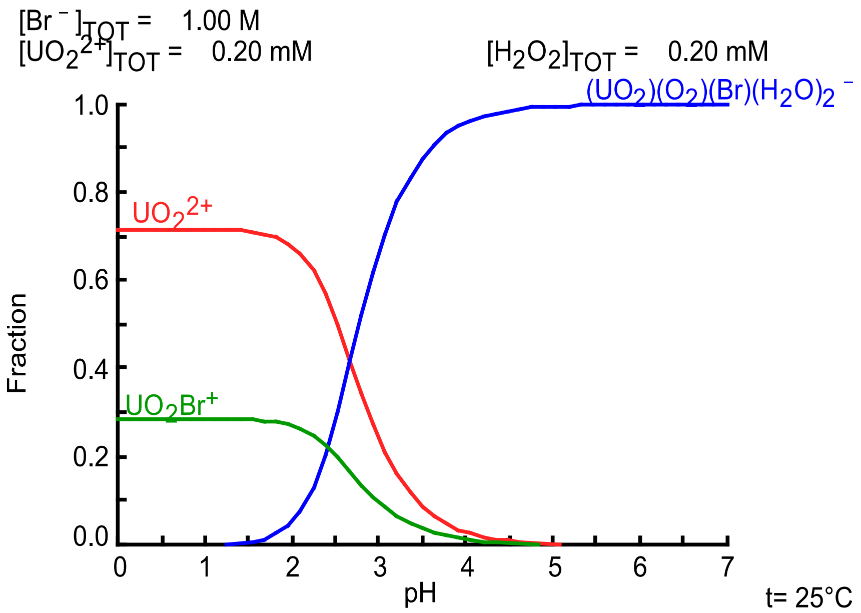

Figure S23. Speciation calculation for 0.2 mM UO_2^{2+} and 0.2 mM H_2O_2 in 1 M Br^- solution as a function of pH based on the estimated stability constant for $(UO_2)(O_2)(Br)(H_2O)_2^-$. The stability constants used for UO_2Br^+ and $(UO_2)(O_2)(Br)(H_2O)_2^-$ are valid at Ionic strength ≈ 5 mol/L.

Table S6. Equilibrium constants of various species used in speciation simulation presented in figure S18 – S23.[1][18]

Species	equilibrium constants[1]
$(UO_2)_2(OH)_2^{2+}$	-5.62
$(UO_2)_2OH^{3+}$	-2.7
$(UO_2)_3(OH)_4^{2+}$	-11.9
$(UO_2)_3(OH)^{5+}$	-15.55
$(UO_2)_3(OH)_7^{-}$	-32.2
$(UO2)_4(OH)_7^{+}$	-21.9
$UO_2(OH)_2$	-12.15
$UO_2(OH)_3^{-}$	-20.25
$UO_2(OH)_4^{2-}$	-32.4
UO_2Cl^+	0.176 (I ≈ 5 mol/L)
UO_2Br^+	-0.4 (I ≈ 5 mol/L)
UO_2OH^+	-5.25
$UO_2(OH)_2.H_2O$	-4.81
$UO_2(O_2)(OH)^-$	-14.16
$(UO_2)(O_2)(Cl)(H_2O)_2^-$	-0.77
$(UO_2)(O_2)(Br)(H_2O)_2^-$	-1.4

References

(1) Grenthe, I.; Fuger, J.; Konings, R. J. M.; Lemire, R. J.; Muller, A. B.; Wanner, H.; Forest, I. CHEMICAL THERMODYNAMICS OF URANIUM. **2004**.

(2) 1. Medusa - Computer Program for Calculating the Composition of Equilibrium Mixtures by I. Puigdomenech, Freely Available from Https://Www.Kth.Se/Che/Medusa.

(3) Nguyen-Trung, C.; Begun, G. M.; Palmer, D. Aqueous Uranium Complexes. 2. Raman Spectroscopic Study of the Complex Formation of the Dioxouranium(VI) Ion with a Variety of Inorganic and Organic Ligands. *Inorg. Chem.* **1992**, *32*, 5280-5287.

(4) Lu, G.; Forbes, T. Z.; Haes, A. J. Evaluating Best Practices in Raman Spectral Analysis for Uranium Speciation and Relative Abundance in Aqueous Solutions. *Anal. Chem.* **2016**, *88*, 773–780. https://doi.org/10.1021/ACS.ANALCHEM.5B03038/SUPPL_FILE/AC5B03038_SI_001.PDF.

(5) Brooker, M. H.; Huang, C. B.; Sylwestrowicz, J. Raman Spectroscopic Studies of Aqueous Uranyl Nitrate and Perchlorate Systems. *J. Inorg. Nucl. Chem.* **1980**, *42*, 1431–1440. https://doi.org/10.1016/0022-1902(80)80109-6.

(6) Lu, G.; Haes, A. J.; Forbes, T. Z. Detection and Identification of Solids, Surfaces, and Solutions of Uranium Using Vibrational Spectroscopy. *Coordination Chemistry Reviews*. **2018**, *374*, 314–344. https://doi.org/10.1016/j.ccr.2018.07.010.

(7) Colmenero, F.; Bonales, L. J.; Cobos, J.; Timón, V. Study of the Thermal Stability of Studtite by in Situ Raman Spectroscopy and DFT Calculations. *Spectrochim. Acta - Part A Mol. Biomol. Spectrosc.* **2017**, *174*, 245–253. https://doi.org/10.1016/j.saa.2016.11.040.

(8) Thompson, N. B. A.; Frankland, V. L.; Bright, J. W. G.; Read, D.; Gilbert, M. R.; Stennett, M. C.; Hyatt, N. C. The Thermal Decomposition of Studtite: Analysis of the Amorphous Phase. *J. Radioanal. Nucl. Chem.* **2021**, *327*, 1335–1347. https://doi.org/10.1007/S10967-021-07611-4/FIGURES/8.

(9) Fairley, M.; Myers, N. M.; Szymanowski, J. E. S.; Sigmon, G. E.; Burns, P. C.; Laverne, J. A. Stability of Solid Uranyl Peroxides under Irradiation. *Inorg. Chem.* **2019**, *58*, 14112–14119. https://doi.org/10.1021/ACS.INORGCHEM.9B02132/SUPPL_FILE/IC9B02132_SI_001.PDF.

(10) Bastians, S.; Crump, G.; Griffith, W. P.; Withnall, R. Raspite and Studtite: Raman Spectra of Two Unique Minerals. *J. Raman Spectrosc.* **2004**, *35*, 726–731. https://doi.org/10.1002/JRS.1176.

(11) Sarrasin, L.; Miro, S.; Jégou, C.; Tribet, M.; Broudic, V.; Marques, C.; Peuget, S. Studtite Formation Assessed by Raman Spectroscopy And18O Isotopic Labeling during the Oxidative Dissolution of a MOX Fuel. *J. Phys. Chem. C.* **2021**, *125*, 19209–19218. https://doi.org/10.1021/ACS.JPCC.1C04392/ASSET/IMAGES/ACS.JPCC.1C04392.SOCIAL.JPEG_V03.

(12) Thompson; Nba; Frankland; VL; Bright; JWG; Read; Gilbert; MR; Stennett; MC; Hyatt; NC. The Thermal Decomposition of Studtite: Analysis of the Amorphous Phase. *J. Radioanal. Nucl. Chem.* **2021**, *327*, 1335-1347.https://doi.org/10.1007/s10967-021-07611-4.

(13) Vacque, V.; Sombret, B.; Huvenne, J. P.; Legrand, P.; Suc, S. Characterisation of the O-O Peroxide Bond by Vibrational Spectroscopy. *Spectrochim. Acta - Part A Mol. Spectrosc.* **1997**, *53*, 55–66. https://doi.org/10.1016/s1386-1425(97)83009-0.

(14) Moreno, T.; Morán López, M. A.; Huerta Illera, I.; Piqueras, C. M.; Sanz Arranz, A.; García Serna, J.; Cocero, M. J. Quantitative Raman Determination of Hydrogen Peroxide Using the Solvent as Internal Standard: Online Application in the Direct Synthesis of Hydrogen Peroxide. *Chem. Eng. J.* **2011**, *166*, 1061–1065. https://doi.org/10.1016/J.CEJ.2010.11.068.

(15) Ramírez-Cedeño, M. L.; Gaensbauer, N.; Félix-Rivera, H.; Ortiz-Rivera, W.; Pacheco-Londoño, L.; Hernández-Rivera, S. P. Fiber Optic Coupled Raman Based Detection of Hazardous Liquids Concealed in Commercial Products. *Int. J. Spectrosc.* **2012**, *2012*, 1–7. https://doi.org/10.1155/2012/463731.

(16) Quilès, F.; Burneau, A. Infrared and Raman Spectroscopic Study of Uranyl Complexes: Hydroxide and Acetate Derivatives in Aqueous Solution. *Vib. Spectrosc.* **1998**, *18*, 61–75. https://doi.org/10.1016/S0924-2031(98)00040-X.

(17) Buck, E. C.; Douglas, M.; Mcnamara, B. K.; Hanson, B. D. Possible Incorporation of Neptunium in Uranyl (VI) Alteration Phases. **2003**.

(18) Soderholm, L.; Skanthakumar, S.; Wilson, R. E. Structural Correspondence between Uranyl Chloride Complexes in Solution and Their Stability Constants. *J. Phys. Chem. A* **2011**, *115*, 4959–4967. https://doi.org/10.1021/jp111551t.
